# Supplementary material for: Commercially Available Cell-Free Permeability Tests for Industrial Drug Development: Increased Sustainability through Reduction of In Vivo Studies
Source: Pharmaceutics. 2023 Feb 9;15(2):592. doi: 10.3390/pharmaceutics15020592 (PMC9964961; doi:10.3390/pharmaceutics15020592)
Supplement: Supplementary file 1 [file pharmaceutics-15-00592-s001.zip › pharmaceutics-2125201-supplementary.pdf]

Supplementary Material

Product overview of the 10 largest pharmaceutical companies:

BCS classification:
BCS1: high solubility, high permeability
BCS2: low solubility, high permeability
BCS3: high solubility, low permeability
BCS4: low solubility, low permeability
BCS0: No information on BCS class available or solubility/permeability data inconclusive

Pfizer products (https://www.pfizer.com/products):

1. ACCUPRIL® Oral BCS1 1991 90s1

   (quinapril HCI)

   https://www.accessdata.fda.gov/drugsatfda_docs/label/2009/019885s028lbl.pdf

2. ACCURETIC® **Oral** BCS0 1999 90s0

   (quinapril HCl/hydrochlorothiazide)

   https://www.accessdata.fda.gov/drugsatfda_docs/label/2009/019885s028lbl.pdf

3. ACETYLCYSTEINE

   (acetylcysteine solution)

4. ADENOSINE

   (adenosine)

5. ALDACTAZIDE® **Oral BCS2 1978 70s2**

   (spironolactone and hydrochlorothiazide)

   https://www.accessdata.fda.gov/drugsatfda_docs/label/2008/012151s062lbl.pdf

6. ALDACTONE® **Oral BCS2 1960 60s2**

   (spironolactone)

   https://www.accessdata.fda.gov/drugsatfda_docs/label/2008/012151s062lbl.pdf

7. ALFENTANIL INJECTION
8. ALTACE® **Oral BCS0 1991 90s0**

(ramipril)

9. AMIDATE™

(etomidate)

10. AMINOCAPROIC ACID
11. AMINOPHYLLINE

(aminophylline)

12. AMIODARONE HYDROCHLORIDE Oral BCS0 1985 80s0
13. AMMONIUM CHLORIDE
14. AMPICILLIN AND SULBACTAM
15. AMPICILLIN FOR INJECTION

Ampicillin Sodium

16. ARGATROBAN

(argatroban injection, solution)

17. AROMASIN® Oral BCS4 1999 90s4

(exemestane)

https://www.accessdata.fda.gov/drugsatfda_docs/label/1999/20753LBL.PDF

18. ARTHROTEC® Oral BCS2 1997 90s2

(diclofenac sodium/misoprostol)

https://www.ema.europa.eu/en/documents/referral/diclofenac-article-29-referral-assessment-report_en.pdf

https://www.ema.europa.eu/en/documents/outside-eu-assessment-report/hemoprostol-public-assessment-report_en.pdf

19. ATGAM®

(lymphocyte immune globulin, anti-thymocyte globulin [equine] sterile solution)

20. ATRACURIUM BESYLATE

(atracurium besylate)

21. ATROPINE SULFATE
22. AZITHROMYCIN
23. AZTREONAM

(aztreonam)

24. AZULFIDINE® Oral BCS2 BCS4 1950 50s4

(sulfasalazine, USP)

https://www.pfizer.ca/sites/default/files/202011/Salazopyrin_PM_E_19Oct2020_239257.pdf

25. BAVENCIO

(avelumab)

26. BENEFIX®

(coagulation factor IX (recombinant))

27. BESPONSA™ for injection

(inotuzumab ozogamicin)

28. BICILLIN® C-R

(penicillin G benzathine and penicillin G procaine injectable suspension)

29. BICILLIN® L-A

(penicillin G benzathine)

30. BIVALIRUDIN

(bivalirudin)

31. BLEOMYCIN
32. BORTEZOMIB

(bortezomib)

33. BOSULIF® Oral BCS4 2012-4 10s4

(bosutinib)

https://www.accessdata.fda.gov/drugsatfda_docs/nda/2012/203341orig1s000clinpharmr.pdf

34. BRAFTOVI® capsules Oral BCS2 2018-2 10s2

(encorafenib)

https://www.ema.europa.eu/en/documents/assessment-report/braftovi-epar-public-assessment-report_en.pdf

35. BUMETANIDE

(bumetanide)

36. BUPIVACAINE

(bupivacaine hydrochloride)

37. BUPRENORPHINE HYDROCHLORIDE

(buprenorphine hydrochloride)

38. BUSULFAN

(busulfan)

39. BUTORPHANOL TARTRATE

(butorphanol tartrate)

40. CADUET® Oral BCS2 2004-2 00s2

(amlodipine besylate/atorvastatin calcium)

https://www.accessdata.fda.gov/drugsatfda_docs/nda/2018/210045Orig1s000ChemR.pdf

https://www.accessdata.fda.gov/drugsatfda_docs/label/2009/020702s057lbl.pdf

41. CALAN® Oral BCS1 1984 80s1

(verapamil hydrochloride)

https://www.accessdata.fda.gov/drugsatfda_docs/label/2016/018925s010lbl.pdf

42. CALCIUM CHLORIDE

(calcium chloride)

43. CAMPTOSAR®

(irinotecan HCI injection)

44. CARBOCAINE

(mepivacaine)

45. CARBOPLATIN

(carboplatin)

46. CARDURA® Oral BCS0 1990 90s0

(doxazosin mesylate)

47. CARDURA® XL Oral BCS0 1990 90s0

(doxazosin mesylate extended release)

48. CAVERJECT®

(alprostadil injection, powder, lyophilized, for solution)

49. CEFAZOLIN

(cefazolin)

50. CEFEPIME

(Cefepime)

51. CEFOBID

(cefoperazone sodium)

52. CEFTRIAXONE

(ceftriaxone)

53. CELEBREX Oral BCS2 1998 90s2

(celecoxib capsules)

https://www.ema.europa.eu/en/documents/scientific-discussion/onsenal-epar-scientific-discussion_en.pdf

54. CELONTIN® Oral BCS0 1957 50s0

(methsuximide capsules)

55. CEREBYX®

(fosphenytoin sodium injection)

56. CHANTIX® Oral BCS1 2006-1 00s1

(varenicline)

https://www.accessdata.fda.gov/drugsatfda_docs/nda/2006/021928_s000_Chantix_ChemR.pdf

57. CHROMIUM

(chromic chloride)

58. CIBINQO™ Oral BCS2 2022-2 20s2

(abrocitinib)

https://www.ema.europa.eu/en/documents/assessment-report/cibinqo-epar-public-assessment-report_en.pdf

59. CIPROFLOXACIN

(ciprofloxacin)

60. CISATRACURIUM BESYLATE

(cisatracurium besylate)

61. CLEOCIN

(clindamycin)

62. COLESTID®

(micronized colestipol hydrochloride)

63. COMIRNATY®

(COVID-19 Vaccine, mRNA)

64. COPPER

(cupric chloride)

65. CORLOPAM®

(fenoldopam mesylate)

66. CORTEF® Oral BCS2 1952 50s2

(hydrocortisone tablets)

https://ec.europa.eu/health/documents/community-register/2011/20111103110069/anx_110069_en.pdf

67. CORTISPORIN®

(neomycin and polymyxin B sulfates, and hydrocortisone acetate)

68. CORVERT®

(ibutilide fumarate injection)

69. CORZIDE® Oral BCS0

(nadolol and bendroflumethiazide)

70. COVERA-HS® Oral BCS1 1984 80s1

(verapamil hydrochloride extended-release)

https://www.accessdata.fda.gov/drugsatfda_docs/label/2016/018925s010lbl.pdf

71. CUTAQUIG®

(Immune Globulin Subcutaneous (Human) - hipp), 16.5% solution

72. CYKLOKAPRON®

(tranexamic acid injection)

73. CYTARABINE

(cytarabine)

74. CYTOMEL® Oral BCS0 1956 50s0

(liothyronine sodium)

75. CYTOTEC® Oral BCS1 1988 80s1

(misoprostol)

https://www.ema.europa.eu/en/documents/outside-eu-assessment-report/hemoprostol-public-assessment-report_en.pdf

76. DACARBAZINE

(dacarbazine)

77. DAPTOMYCIN

(daptomycin)

78. DAURISMO Tablets Oral BCS4 2018-4 10s4

(glasdegib)

https://www.ema.europa.eu/en/documents/assessment-report/daurismo-epar-public-assessment-report_en.pdf

79. DAYPRO ALTA™ Oral BCS2 1992 90s2

(oxaprozin potassium)

https://www.accessdata.fda.gov/drugsatfda_docs/label/2007/18841s022lbl.pdf

80. DAYPRO® Oral BCS2 1992 90s2

(oxaprozin)

https://www.accessdata.fda.gov/drugsatfda_docs/label/2007/18841s022lbl.pdf

81. DEFEROXAMINE MESYLATE

(deferoxamine mesylate)

82. DEMEROL™

(meperidine hydrochloride)

83. DEPO-MEDROL®

(methylprednisolone acetate injectable suspension)

84. DEPO-PROVERA

(medroxyprogesterone acetate injectable suspension)

85. DEPO-SUBQ PROVERA 104®

(medroxyprogesterone acetate injectable suspension)

86. DEPO®-ESTRADIOL

(estradiol cypionate injection)

87. DEPO®-TESTOSTERONE

(testosterone cypionate injection)

88. DETROL® Oral BCS1 1998 90s1

(tolterodine tartrate)

https://www.accessdata.fda.gov/drugsatfda_docs/label/2009/020771s022lbl.pdf

89. DEXTROSE

(dextrose)

90. DIABINESE® Oral BCS2 1998 90s2

(chlorpropamide)

https://www.accessdata.fda.gov/drugsatfda_docs/label/2008/011641s064lbl.pdf

91. DIAZEPAM

(diazepam)

92. DIDREX Oral BCS1 1960 60s1

(benzphetamine hydrochloride)

https://www.accessdata.fda.gov/drugsatfda_docs/label/2010/012427s026lbl.pdf

93. DIFLUCAN® Oral BCS0 1988 80s0

(fluconazole)

94. DILANTIN® Oral BCS2 1953 50s2

(phenytoin oral suspension, phenytoin, and extended phenytoin sodium)

https://www.accessdata.fda.gov/drugsatfda_docs/label/2016/008762s057s058lbl.pdf

95. DILTIAZEM HYDROCHLORIDE

(diltiazem hydrochloride)

96. DOBUTAMINE

(dobutamine)

97. DOCETAXEL INJECTION
98. DOPAMINE

(dopamine)

99. DOSTINEX® Oral BCS2 BCS4 1996 90s4

(cabergoline)

https://www.accessdata.fda.gov/drugsatfda_docs/label/2014/020664s013lbl.pdf

100. DOXERCALCIFEROL

(doxercalciferol)

101. DOXORUBICIN HYDROCHLORIDE

(doxorubicin hydrochloride)

102. DROPERIDOL

(droperidol)

103. DUAVEE® Oral BCS0

(conjugated estrogens/bazedoxifene)

104. EFFEXOR® XR Oral BCS1 1997 90s1

(venlafaxine hydrochloride)

https://www.accessdata.fda.gov/drugsatfda_docs/label/2005/020699s059lbl.pdf

105. ELELYSO™

(taliglucerase alfa)

106. ELIQUIS Oral BCS3 2012-3 10s3

(apixaban)

https://www.ema.europa.eu/en/documents/scientific-guideline/apixaban-film-coated-tablet-25-5-mg-product-specific-bioequivalence-guidance_en.pdf

107. ELLENCE®

(epirubicin hydrochloride)

108. EMCYT® Oral BCS1 BCS3 1981 80s1

(estramustine phosphate sodium)

https://cdn.pfizer.com/pfizercom/products/uspi_emcyt.pdf

109. ENALAPRILAT

(enalaprilat)

110. EPINEPHRINE

(epinephrine)

111. ERAXIS™

(anidulafungin)

112. Ertapenem

(Ertapenem)

113. ERYTHROCIN™ LACTOBIONATE

(erythromycin lactobionate)

114. ESTRING®

(estradiol vaginal ring)

115. EUCRISA™

(crisaborole)

116. FELDENE® Oral BCS2 1982 80s2

(piroxicam)

https://www.accessdata.fda.gov/drugsatfda_docs/label/2006/018147s029lbl.pdf

117. FENTANYL CITRATE

(fentanyl citrate)

118. FLAGYL® Oral BCS0

(metronidazole)

119. FLUCONAZOLE

(fluconazole)

120. FOSCAVIR®

(foscarnet sodium)

121. FRAGMIN®

(dalteparin sodium injection)

122. FUROSEMIDE

(furosemide)

123. GELFILM®

(gelfilm absorbable film)

124. GELFOAM® and GEL-FLOW NT

(absorbable gelatin)

125. GEMCITABINE

(gemcitabine)

126. GENOTROPIN

(somatropin [rDNA origin])

127. GENTAMICIN SULFATE

(gentamicin sulfate)

128. GEODON Oral BCS4 2001 00s4

(ziprasidone HCl)

https://www.accessdata.fda.gov/drugsatfda_docs/nda/2002/20-919_Geodon_biopharmr.pdf

129. GLUCOTROL® Oral BCS2 1994 90s2

(glipizide)

https://www.accessdata.fda.gov/drugsatfda_docs/label/2008/017783s019lbl.pdf

130. GLYNASE® PRESTAB® Oral BCS0

(micronized glyburide)

131. GLYSET® Oral BCS1 1999 90s1

(miglitol)

https://www.accessdata.fda.gov/drugsatfda_docs/label/2012/020682s010lbl.pdf

132. HALCION® Oral BCS2 1982 80s2

(triazolam)

https://www.accessdata.fda.gov/drugsatfda_docs/label/2019/017892s050lbl.pdf

133. HEMABATE®

(carboprost tromethamine)

134. HEPARIN SODIUM INJECTION

(heparin sodium)

135. HETASTARCH

(hetastarch)

136. HEXTEND

(hetastarch)

137. HUMATIN

(paromomycin sulfate)

138. HYDROMORPHONE HYDROCHLORIDE

(hydromorphone hydrochloride)

139. IBRANCE® Oral BCS2 2015-2 10s2

(palbociclib)

https://www.ema.europa.eu/en/documents/scientific-guideline/draft-palbociclib-hard-capsule-75-mg-100-mg-125-mg-product-specific-bioequivalence-guidance_en-0.pdf

140. IDAMYCIN®

(idarubicin hydrochloride)

141. INDOMETHACIN

(indomethacin)

142. INFLECTRA®

(infliximab-dyyb)

143. INLYTA® Oral BCS2 2012-2 10s2

(axitinib)

https://www.accessdata.fda.gov/drugsatfda_docs/nda/2012/202324orig1s000clinpharmr.pdf

144. INSPRA Oral BCS4 2002-4 00s4

(eplerenone)

https://www.accessdata.fda.gov/drugsatfda_docs/label/2016/021437s013lbl.pdf

145. INTAL® Nebulizer

(cromolyn sodium)

146. IRINOTECAN HYDROCHLORIDE

(irinotecan hydrochloride)

147. IXIFI™ for injection, for Intravenous Use

(infliximab-qbtx)

148. KERYDIN®

(tavaborole)

149. KETAMINE HYDROCHLORIDE

(ketamine hydrochloride)

150. KETOROLAC TROMETHAMINE

(ketorolac tromethamine)

151. LABETALOL HYDROCHLORIDE

(labetalol hydrochloride)

152. LEVETIRACETAM

(levetiracetam)

153. LEVOFLOXACIN

(levofloxacin)

154. LEVOPHED

(norepinephrine)

155. LEVOXYL® Oral BCS0

(levothyroxine sodium)

156. LIDOCAINE HYDROCHLORIDE

(lidocaine hydrochloride)

157. LINCOCIN®

(lincomycin)

158. LINEZOLID

(linezolid)

159. LIPITOR® Oral BCS4 1996 90s4

(atorvastatin calcium)

https://www.accessdata.fda.gov/drugsatfda_docs/label/2009/020702s057lbl.pdf

160. LO/OVRAL® Oral BCS0 1979 70s0

(norgestrel and ethinyl estradiol)

161. Lomotil® Oral BCS0

(diphenoxylate hydrochloride with atropine sulfate)

162.LONITEN® Oral BCS0 1979 70s0

(minoxidil tablets)

163.LOPID® Oral BCS2 1982 80s2

(gemfibrozil)

https://www.accessdata.fda.gov/drugsatfda_docs/label/2016/018422s055lbl.pdf

164.LORAZEPAM

(lorazepam)

165.LORBRENA® Oral BCS4 2018-4 10s4

(lorlatinib)

https://www.accessdata.fda.gov/drugsatfda_docs/nda/2018/210868Orig1s000ChemR.pdf

166.LOW MOLECULAR WEIGHT DEXTRAN

(dextran 40)

167.LYRICA® and LYRICA®CR Oral BCS1 2004-1 00s1

(pregabalin) and (pregabalin extended release tablets)

https://www.ema.europa.eu/en/documents/assessment-report/pregabalin-zentiva-epar-public-assessment-report_en.pdf

168.MAGNESIUM SULFATE

(magnesium sulfate)

169.MANGANESE CHLORIDE

(manganese chloride)

170.MANNITOL

(mannitol)

171.MARCAINE

(bupivacaine hydrochloride)

172. MAXIPIME

(cefepime hydrochloride)

173. MEDROL® Oral BCS2 1957 50s2

(methylprednisolone)

https://www.accessdata.fda.gov/drugsatfda_docs/label/2018/011153s075lbl.pdf

174. MEKTOVI® tablets Oral BCS4 2018-4 10s4

(binimetinib)

https://www.accessdata.fda.gov/drugsatfda_docs/label/2018/210498lbl.pdf

175. MENEST® Oral BCS0

(esterified estrogens)

176. MEPIVACAINE

(mepivacaine)

177. MEROPENEM

(Meropenem)

178. MERREM® I.V.

(meropenem for injection)

179. METHOTREXATE

(methotrexate)

180. METOCLOPRAMIDE HYDROCHLORIDE

(metoclopramide)

181. METOPROLOL

(metoprolol)

182. METRONIDAZOLE

(metronidazole)

183. MICRONASE® Oral BCS0 1984 80s0

(glyburide)

184. MIDAZOLAM

(midazolam)

185. MILRINONE LACTATE

(milrinone lactate)

186. MINIPRESS® Oral BCS0 1988 80s0

(prazosin hydrochloride)

187. MITOXANTRONE HYDROCHLORIDE

(mitoxantrone hydrochloride)

188. MORPHINE SULFATE

(Morphine Sulfate)

189. MULTI-VITAMIN INFUSION
190. MYCOBUTIN® Oral BCS0 1992 90s0

(rifabutin)

191. MYFEMBREE® Tablets

(relugolix, estradiol, and norethindrone acetate)

192. MYLOTARG™

(gemtuzumab ozogamicin for injection)

193. Nadolol Tablets Oral BCS3 1979 70s3

(nadolol)

doi.org/10.1021/mp070028i

194. NALBUPHINE HYDROCHLORIDE

(nalbuphine hydrochloride)

195. NALOXONE HYDROCHLORIDE

(naloxone hydrochloride)

196.NARDIL® Oral BCS0 1961 60s0

(phenelzine sulfate)

197.NAVANE® Oral BCS0

(thiothixene)

198.NEOSPORIN® G.U. Irrigant

(neomycin sulfate – polymyxin B sulfate)

199.NEOSPORIN® Ophthalmic Solution

(neomycin and polymyxin B sulfates, and gramicidin)

200.NEURONTIN® Oral BCS3 2000-3 00s

(gabapentin)

https://www.fda.gov/media/108504/download

201.NICOTROL® Inhaler

(nicotine)

202.NICOTROL® NS

(nicotine)

203.NIPENT™

(pentostatin)

204.NITROSTAT®

(nitroglycerin)

205.NIVESTYM™

(filgrastim-aafi)

206.NORMINEST® FE Tablets and NORQUEST® FE Tablets Oral BCS0

norethindrone and ethinyl estradiol

207.NORPACE® and NORPACE® CR Oral BCS1 1977 70s1

(disopyramide phosphate)

https://www.pfizermedicalinformation.com/en-us/norpace/description

208.North American Coral Snake Antivenin

(Equine)

209.NORVASC® Oral BCS1 1987 80s1

(amlodipine besylate)

https://www.accessdata.fda.gov/drugsatfda_docs/nda/2018/210045Orig1s000ChemR.pdf

210.NYVEPRIA™

(pegfilgrastim-apgf)

211.OCTAGAM

Immune Globulin Intravenous (Human)

212.OGEN® Oral BCS1 BCS3 1977-3 70s3

(estropipate)

https://labeling.pfizer.com/ShowLabeling.aspx?id=668

213.ONDANSETRON

(ondansetron)

214.ORGOVYX® Tablets Oral BCS4 2020-4 20s4

(relugolix)

https://www.accessdata.fda.gov/drugsatfda_docs/nda/2020/214621Orig1s000ChemR.pdf

215.OXALIPLATIN

(oxaliplatin)

216.PACLITAXEL

(paclitaxel)

217. Palonosetron Hydrochloride

(palonosetron hydrochloride)

218. PAMIDRONATE DISODIUM

(pamidronate disodium)

219. PANCURONIUM BROMIDE

(pancuronium bromide)

220. PANZYGA

(immune globulin intravenous, human - ifas)

221. PARICALCITOL

(paricalcitol)

222. PAXLOVID™ Oral BCS4 2021-4 20s4

(nirmatrelvir and ritonavir)

https://www.accessdata.fda.gov/drugsatfda_docs/nda/2010/022417s000_ChemR.pdf

223. PEMETREXED INJECTION
224. PENICILLIN G PROCAINE

(penicillin g procaine)

225. Pfizer-BioNTech COVID-19 Vaccine

(also known as BNT162b2)

226. PFIZERPEN®

(penicillin G potassium)

227. PHOSPHOLINE IODIDE®

(echothiophate iodide)

228. PIPERACILLIN AND TAZOBACTAM

(piperacillin and tazobactam)

229. PLEGISOL®

(potassium chloride, sodium chloride, calcium chloride, and magnesium chloride)

230.POTASSIUM ACETATE

(potassium acetate)

231.POTASSIUM CHLORIDE

(potassium chloride)

232.POTASSIUM PHOSPHATES

(potassium phosphates)

233.PRAZOSIN HYDROCHLORIDE Oral BCS0 1988 80s0

(PRAZOSIN HYDROCHLORIDE)

234.PRECEDEX™

(dexmedetomidine hydrochloride)

235.PREMARIN® Oral BCS0

(conjugated estrogens)

236.PREMPRO®/PREMPHASE® Oral BCS0

(conjugated estrogens/medroxyprogesterone acetate)

237.PREPIDIL®

(dinoprostone)

238.PREVNAR 13

(pneumococcal 13-valent conjugate vaccine [diphtheria CRM197 Protein])

239.Prevnar 20™

(Pneumococcal 20-valent Conjugate Vaccine)

240.PRISTIQ® Oral BCS1 2008-1 00s1

(desvenlafaxine)

https://www.accessdata.fda.gov/drugsatfda_docs/label/2012/021992s030lbl.pdf

241. PROCAINAMIDE HYDROCHLORIDE

(procainamide hydrochloride)

242. PROCARDIA® Oral BCS2 1981 80s2

(nifedipine)

https://www.accessdata.fda.gov/drugsatfda_docs/label/2011/020198s023lbl.pdf

243. PROPOFOL

(propofol)

244. PROSTIN E2®

(dinoprostone)

245. PROSTIN VR PEDIATRIC®

(alprostadil)

246. PROTONIX® Oral BCS3 2000-3 00s3

(pantoprazole sodium)

https://ec.europa.eu/health/documents/community-register/2010/2010033072510/anx_72510_en.pdf

247. PROVERA® Oral BCS2 1959 50s2

(medroxyprogesterone acetate)

https://extranet.who.int/pqweb/sites/default/files/RH074part6v02.pdf

248. PSORCON/FLORONE

diflorasone diacetate

249. QUELICIN™

(succinylcholine chloride)

250. R-GENE® 10

(arginine hydrochloride)

251. RAPAMUNE® Oral BCS2 1999 90s2

(sirolimus)

https://www.ema.europa.eu/en/documents/scientific-guideline/sirolimus-coated-tablets-05-1-2-mg-oral-solution-1-mg/ml-product-specific-bioequivalence-guidance_en.pdf

252. RELPAX® Oral BCS1 2002-1 00s1

(eletriptan HBr)

https://www.accessdata.fda.gov/drugsatfda_docs/label/2011/021016s018lbl.pdf

253. RETACRIT Injection

(epoetin alfa-epbx)

254. REVATIO® Oral BCS1 1998 90s1

(sildenafil)

https://www.ema.europa.eu/en/documents/assessment-report/sildenafil-teva-epar-public-assessment-report_en.pdf

255. ROCURONIUM BROMIDE

(rocuronium bromide)

256. ROPIVACAINE HYDROCHLORIDE

(ropivacaine hydrochloride)

257. RUXIENCE™

(rituximab-pvvr)

258. SEGLUROMET™ Tablets Oral BCS3 2017-3 10s3

(ertugliflozin and metformin HCl)

259. SEPTRA® Oral BCS0

(trimethoprim and sulfamethoxazole)

260. SILVADENE®

(silver sulfadiazine)

261. SINEQUAN® Oral BCS1 1969 60s1

(doxepin hydrochloride)

https://www.accessdata.fda.gov/drugsatfda_docs/nda/2010/022036Orig1s000ClinPharmR.pdf

262. SKELAXIN® Oral BCS2 1962 60s2

(metaxalone)

https://www.accessdata.fda.gov/drugsatfda_docs/label/2018/013217s057lbl.pdf

263. SODIUM ACETATE

(sodium acetate)

264. SODIUM BICARBONATE

(sodium bicarbonate)

265. SODIUM CHLORIDE

(sodium chloride)

266. SODIUM LACTATE

(sodium lactate)

267. SODIUM PHOSPHATES

(monobasic sodium phosphate and dibasic sodium phosphate)

268. SOLU-CORTEF®

(hydrocortisone sodium succinate)

269. SOLU-MEDROL®

(methylprednisolone sodium succinate)

270. SOMAVERT®

(pegvisomant)

271. SONATA® CIV Oral BCS2 1999 90s2

(zaleplon)

https://www.accessdata.fda.gov/drugsatfda_docs/label/2013/020859s013lbl.pdf

272. STEGLATRO™ Oral BCS1 2017-1 10s1

(ertugliflozin)

https://www.ema.europa.eu/en/documents/assessment-report/steglatro-epar-public-assessment-report_en.pdf

273. STEGLUJAN™ Tablets Oral BCS1 2017-1 10s1

(ertugliflozin and sitagliptin)

https://www.ema.europa.eu/en/documents/assessment-report/steglatro-epar-public-assessment-report_en.pdf

https://www.ema.europa.eu/en/documents/scientific-guideline/sitagliptin-film-coated-tablets-25-50-100-mg-product-specific-bioequivalence-guidance_en.pdf

274. SUFENTANIL CITRATE

(sufentanil citrate)

275. SUTENT® Oral BCS3 2006-3 00s3

(sunitinib malate)

https://www.ema.europa.eu/en/documents/scientific-guideline/sunitinib-hard-capsules-125-25-375-50-mg-product-specific-bioequivalence-guidance_en.pdf

276. SYNAREL®

(nafarelin acetate)

277. SYNERCID® I.V.

(quinupristin / dalfopristin)

278. TACROLIMUS

(Tacrolimus)

279. TALZENNA™ Oral BCS4 2018-4 10s4

(talazoparib) Capsules

https://www.accessdata.fda.gov/drugsatfda_docs/nda/2018/211651Orig1s000ChemR.pdf

280. TAPAZOLE® Oral BCS1 1950 50s1

(methimazole)

https://www.accessdata.fda.gov/drugsatfda_docs/label/2012/040350s016lbl.pdf

281. TAZICEF®

(ceftazidime)

282. TESSALON® Oral BCS0 1958 50s0

(benzonatate)

283. TESTOSTERONE CYPIONATE

(testosterone cypionate)

284. THAM
285. THEOPHYLLINE

(theophylline)

286. THROMBI-GEL®

(thrombin / gelatin foam hemostat)

287. THROMBI-PAD®

(3x3 hemostatic pad)

288. THROMBIN-JMI®

(thrombin, topical, bovine origin)

289. TICOVAC

(Tick-Borne Encephalitis Vaccine)

290. TIKOSYN® Oral BCS2 1999 90s

(dofetilide)

https://www.accessdata.fda.gov/drugsatfda_docs/label/2016/207058Orig1s000lbl.pdf

291. TOBRAMYCIN

(tobramycin)

292. TOPOTECAN

(topotecan)

293. TORISEL®

(temsirolimus)

294. TOVIAZ™ Oral BCS0 2008-0 00s0

(fesoterodine fumarate)

295. TPN ELECTROLYTES

(sodium chloride, calcium chloride, potassium chloride, magnesium chloride, and sodium acetate anhydrous)

296. TRAZIMERA for Injection

(trastuzumab-qyyp)

297. TRECATOR® Oral BCS2 1968 60s2

(ethionamide)

https://www.accessdata.fda.gov/drugsatfda_docs/label/2006/013026s024lbl.pdf

298. TRIPHASIL-21® and TRIPHASIL-28® Tablets Oral BCS0

levonorgestrel and ethinyl estradiol - triphasic regimen

299. TROBICIN™

(spectinomycin)

300. TRUMENBA™

(Meningococcal Group B Vaccine)

301. TYGACIL®

(tigecycline)

302. UNASYN®

(ampicillin sodium/sulbactam sodium)

303. Vancomycin Hydrochloride

(vancomycin hydrochloride)

304. VANTIN® Oral BCS0 1998 90s0

(cefpodoxime proxetil)

305. Vecuronium Bromide

(vecuronium bromide for injection)

306. VERAPAMIL HYDROCHLORIDE

(verapamil hydrochloride)

307. VFEND® Oral BCS2 2002-2 00s2

(voriconazole)

https://www.ema.europa.eu/en/documents/scientific-guideline/voriconazole-tablets-50-200-mg-powder-oral-suspension-40-mg/ml-product-specific-bioequivalence-guidance_en.pdf

308. VIAGRA® Oral BCS1 1998 90s1

(sildenafil citrate)

309. VIBRAMYCIN® Oral BCS1

(doxycycline calcium, doxycycline hyclate, doxycycline monohydrate)

https://www.accessdata.fda.gov/drugsatfda_docs/nda/2014/205931Orig1s000ClinPharmR.pdf

310. VINCRISTINE SULFATE

(vincristine sulfate)

311. VIRACEPT® Oral BCS4 2003-4 00s4

(nelfinavir mesylate)

https://www.accessdata.fda.gov/drugsatfda_docs/label/2005/021503s006lbl.pdf

312. VIROPTIC®

(trifluridine)

313. VISTARIL® Oral BCS1 1956 50s1

(hydroxyzine pamoate)

https://www.accessdata.fda.gov/drugsatfda_docs/label/2014/088617Orig1s043,088618Orig1s043,088619Orig1s044lbl.pdf

314. VITAMIN K

(phytonadione)

315. VIZIMPRO® Tablets Oral BCS2 2018-2 10s2

(dacomitinib)

https://www.ema.europa.eu/en/documents/assessment-report/vizimpro-epar-public-assessment-report_en.pdf

316. VYNDAQEL® (tafamidis meglumine) Capsules and VYNDAMAX™ (tafamidis) Capsules Oral BCS0 2019-0 10s0

317. WATER

(water)

318. XALATAN®

(latanoprost)

319. XALKORI® Oral BCS4 2016-4 10s4

(crizotinib)

https://www.ema.europa.eu/en/documents/scientific-guideline/crizotinib-hard-capsules-200-mg-250-mg-product-specific-bioequivalence-guidance_en.pdf

320. XANAX® Oral BCS2 1981 80s2

(alprazolam)

https://www.accessdata.fda.gov/drugsatfda_docs/label/2011/018276s045lbl.pdf

321. XELJANZ® Oral BCS3 2012-3 10s3

(tofacitinib)

https://www.ema.europa.eu/en/documents/assessment-report/xeljanz-epar-public-assessment-report_en-0.pdf

322. XTANDI® (enzalutamide) capsules, for o use Oral BCS2 2012-2 10s2

Enzalutamide

https://www.accessdata.fda.gov/drugsatfda_docs/nda/2020/213674Orig1s000ChemR.pdf

323.XYNTHA®

(antihemophilic factor (recombinant))

324.ZARONTIN® Oral BCS1 1960 60s1

(ethosuximide)

325.ZINC

(zinc chloride)

326.ZINECARD®

(dexrazoxane)

327.ZIRABEV Injection

(bevacizumab-bvzr)

328.ZITHROMAX® Oral BCSI BCS3 1991 90s1

(azithromycin)

329.ZMAX® Oral BCS0 1991 90s1

(azithromycin extended release)

330.ZOLEDRONIC ACID

(zoledronic acid)

331.ZOLOFT® Oral BCS2 1999 90s2

(sertraline HCI)

https://www.accessdata.fda.gov/drugsatfda_docs/label/2011/019839s072s075s076,020990s033s036s037lbl.pdf

332.ZOSYN®

(piperacillin and tazobactam)

333.ZYVOX® Oral BCS1 2000-1 00s1

(linezolid)
https://extranet.who.int/pqweb/sites/default/files/documents/BE_linezolid_July2021.pdf

Novartis products:

Novartis products worldwide
https://www.novartis.com/about/products?search_api_fulltext=&sort_by=title&sort_order=DESC&page=9

1. **Adakveo®** crizanlizumab
2. **Afinitor Disperz®/Votubia®** everolimus **oral BCS4 2009-4 00s4**
   https://www.ema.europa.eu/en/documents/scientific-guideline/draft-everolimus-tablets-025-05-075-1mg-25-5-10mg-dispersible-tablets-01-025mg-2-3-5mg-product_en.pdf
3. **Afinitor®/Votubia®** everolimus **oral BCS4 2009-4 00s4**
   https://www.ema.europa.eu/en/documents/scientific-guideline/draft-everolimus-tablets-025-05-075-1mg-25-5-10mg-dispersible-tablets-01-025mg-2-3-5mg-product_en.pdf
4. **Aimovig®** erenumab
5. **Arzerra®** ofatumumab
6. **Azarga®/Azorga®** brinzolamide, timolol
7. **Beovu®** brolucizumab
8. **Cibacen®** benazepril hydrochloride **oral BCS3 1991 90s3**
   https://www.accessdata.fda.gov/drugsatfda_docs/label/2009/020033s038lbl.pdf
9. **Ciprodex®** ciprofloxacin, dexamethasone
10. **Comtan®** entacapone **oral BCS4 1999 90s4**
    https://www.ema.europa.eu/en/documents/assessment-report/entacapone-teva-epar-public-assessment-report_en.pdf
11. **Cosentyx®** secukinumab
12. **Diovan HCT® /Co-Diovan®** valsartan, hydrochlorothiazide **oral BCS0 1998 90s0**
13. Diovan® valsartan oral BCS0 1996 90s0
14. **Duotrav™** travoprost, timolol
15. **Durezol®** difluprednate
16. **Egaten®** triclabendazole **oral BCS2 BCS4 2019-4 10s4**
    https://www.accessdata.fda.gov/drugsatfda_docs/nda/2018/208711Orig1s000ChemR.pdf
17. Entresto® sacubitril, valsartan **oral BCS0 2015-0 10s0**
18. **Eucreas®** vildagliptin, metformin **oral BCS3 2008-3 00s3**
    https://www.ema.europa.eu/en/documents/assessment-report/vildagliptin/metformin-hydrochloride-accord-epar-public-assessment-report_en.pdf
19. **Exelon®** rivastigmine **oral BCS1 BCS3 2000-3 00s3**
20. **Exforge HCT®** valsartan, amlodipine besylate, hydrochlorothiazide **oral BCS0 2009-0 00s0**
21. **Exforge®** valsartan, amlodipine besylate **oral BCS0 2007-0 00s0**

22. Exjade® deferasirox oral BCS2 2005-2 00s2
https://www.accessdata.fda.gov/drugsatfda_docs/nda/2015/206910Orig1s000ClinPharmR.pdf

23. Extavia® interferon beta-1b

24. Farydak® panobinostat oral BCS2 2015-2 10s2
https://www.accessdata.fda.gov/drugsatfda_docs/nda/2015/205353Orig1s000ClinPharmR.pdf

25. Femara® letrozole **oral BCS2 2001-2 00s2**
https://www.accessdata.fda.gov/drugsatfda_docs/label/2007/020726s014lbl.pdf

26. Focalin®dexmethylphenidate HCl, dexmethylphenidate extended rel **oral BCS1 2005-1 00s1**
https://www.ema.europa.eu/en/documents/referral/methylphenidate-hexal-article-29-referral-assessment-report_en.pdf

27. Focalin® XRdexmethylphenidate HCl, dexmethylphenidate extended release) **oral BCS1 2005-1 00s1**

28. Galvus® vildagliptin **oral BCS1 2007-1 00s1**
https://www.ema.europa.eu/en/documents/assessment-report/vildagliptin/metformin-hydrochloride-accord-epar-public-assessment-report_en.pdf

29. Gilenya® fingolimod **oral BCS2 BCS4 2010-4 10s4**
https://www.ema.europa.eu/en/documents/scientific-guideline/fingolimod-capsules-025-05-mg-product-specific-bioequivalence-guidance_en.pdf

30. Gleevec®/Glivec® imatinib mesylate **oral BCS0 2001-0 00s0**
https://www.ema.europa.eu/en/documents/scientific-guideline/imatinib-hard-capsules-50-100-mg-film-coated-tablets-100-400-mg-product-specific-bioequivalence_en.pdf

31. Ilaris® canakinumab

32. Ilevro®/Nevanac® nepafenac

33. Izba® travoprost

34. Jadenu® deferasirox **oral BCS2 2005-2 00s2**
https://www.accessdata.fda.gov/drugsatfda_docs/nda/2015/206910Orig1s000ClinPharmR.pdf

35. **Jakavi®** ruxolitinib **oral BCS1 2011-1 10s1**
https://ec.europa.eu/health/documents/community-register/2012/20120823123254/anx_123254_en.pdf

36. Kesimpta® ofatumumab

37. Kisqali® ribociclib **oral BCS4 2017-4 10s4**
https://www.accessdata.fda.gov/drugsatfda_docs/nda/2017/209092Orig1s000ChemR.pdf

38. Kymriah® tisagenlecleucel-T

39. Lamisil® terbinafine (terbinafine hydrochloride)

40. **Leqvio®** inclisiran
41. **Lescol®** fluvastatin sodium **oral BCS1 1999 90s1**
42. **Lescol® XL** fluvastatin sodium
43. **Lucentis®** ranibizumab
44. **Lutathera®** lutetium Lu 177 dotatate
45. **Luxturna®** voretigene neparvovec
46. **Mayzent®** siponimod **oral BCS2 2019-2 10s2**
https://www.ema.europa.eu/en/documents/variation-report/mayzent-h-c-4712-x-07-epar-assessment-report-variation_en.pdf
47. **Mekinist®** trametinib **oral BCS4 2013-4 10s4**
https://www.ema.europa.eu/en/documents/assessment-report/mekinist-epar-public-assessment-report_en.pdf
48. **Myfortic®** mycophenolic acid (as mycophenolate sodium) **oral BCS**
49. Neoral®/Sandimmune® cyclosporine, USP Modified oral BCS0 1983 80s0
50. **NETSPOT®** gallium Ga 68 dotatate injection
51. **Onbrez® Breezhaler®** indacaterol
52. **Pataday™** olopatadine
53. **Patanol®** olopatadine
54. **Pazeo®** olopatadine
55. **Piqray®** alpelisib **oral BCS2 2019-2 10s2**
56. **Pluvicto™** lutetium Lu 177 vipivotide tetraxetan
57. **Proleukin®** aldesleukin
58. **Promacta®/Revolade®** eltrombopag **oral BCS4 2008-4 00s4**
https://www.accessdata.fda.gov/drugsatfda_docs/label/2008/022291lbl.pdf
59. **Ritalin LA®** methylphenidate HCl modified release **oral BCS1 1955 50s1**
https://www.ema.europa.eu/en/documents/referral/methylphenidate-hexal-article-29-referral-assessment-report_en.pdf
60. **Ritalin®** methylphenidate HCl **oral BCS1 1955 50s1**
https://www.ema.europa.eu/en/documents/referral/methylphenidate-hexal-article-29-referral-assessment-report_en.pdf
61. **Rydapt®** midostaurin **oral BCS2 2017-2 10s2**
https://www.accessdata.fda.gov/drugsatfda_docs/nda/2017/207997Orig1Orig2s000CrossR.pdf
62. **Sandostatin LAR®** octreotide acetate
63. **Sandostatin® SC** octreotide acetate
64. **Scemblix®** asciminib **oral BCS2 BCS4 2021-4 20s4**
https://www.accessdata.fda.gov/drugsatfda_docs/nda/2021/215358Orig1s000,Orig2s000ChemR.pdf
65. **Seebri Breezhaler®** glycopyrronium bromide
66. **Simbrinza®** brinzolamide, brimonidine tartrate

67. Simulect® basiliximab

68. **Stalevo®** carbidopa, levodopa, entacapone **oral BCS4 2003-4 00s4**
https://www.ema.europa.eu/en/documents/assessment-report/entacapone-teva-epar-public-assessment-report_en.pdf

69. Systane® polyethylene glycol 400, propylene glycol

70. Systane® Balance propylene glycol

71. Systane® Hydration polyethylene glycol 400, propylene glycol, hyaluronic acid

72. Systane® Ultra polyethylene glycol 400, propylene glycol

73. Tabrecta® capmatinib **oral BCS2 2020-2 20s2**
https://www.accessdata.fda.gov/drugsatfda_docs/nda/2020/213591Orig1s000MultidisciplineR.pdf

74. **Tafinlar®** dabrafenib **oral BCS2 2013-2 10s2**
https://www.ema.europa.eu/en/documents/assessment-report/tafinlar-epar-public-assessment-report_en.pdf

75. Tasigna® nilotinib **oral BCS4 2007-4 00s4**
https://www.ema.europa.eu/en/documents/scientific-discussion/tasigna-epar-scientific-discussion_en.pdf

76. Tegretol® carbamazepine **oral BCS2 1968 60s2**
10.1016/j.xphs.2021.02.0190022-3549
https://www.fda.gov/media/108504/download

77. TOBI® tobramycin

78. TOBI® Podhaler™ tobramycin

79. Tobradex® tobramycin, dexamethasone

80. Travatan Z® travoprost

81. Travatan® travoprost

82. Travatan® BAK-Free travoprost

83. Trileptal® oxcarbazepine **oral BCS2 2000-2 00s2**
https://www.fda.gov/files/drugs/published/Oxcarbazepine-202810-Clinpharm-PREA.pdf

84. Tyverb® lapatinib **oral BCS2 BCS4 2007-4 00s4**
https://www.ema.europa.eu/en/documents/scientific-guideline/lapatinib-film-coated-tablet-250-mg-product-specific-bioequivalence-guidance-first-version_en.pdf

85. Ultibro® Breezhaler® indacaterol, glycopyrronium bromide

86. Vijoice® alpelisib **oral BCS2 2022-2 20s2**
https://www.accessdata.fda.gov/drugsatfda_docs/nda/2019/212526Orig1s000MultidisciplineR.pdf

87. Votrient® pazopanib **oral BCS2 2009-2 00s2**
https://www.accessdata.fda.gov/drugsatfda_docs/nda/2009/022465s000_ChemR.pdf

88. Xolair® omalizumab

89. Zolgensma® onasemnogene abeparvovec

90. Zometa® zoledronic acid

91. Zortress®/Certican® everolimus **oral BCS2 BCS 4** **2009-4** **00s4**

https://www.ema.europa.eu/en/documents/scientific-guideline/draft-everolimus-tablets-025-05-075-1mg-25-5-10mg-dispersible-tablets-01-025mg-2-3-5mg-product_en.pdf

92. Zykadia® ceritinib **oral BCS4** **2017-4** **10s4**

https://www.accessdata.fda.gov/drugsatfda_docs/nda/2014/205755orig1s000clinpharmr.pdf

AbbVie products (https://www.abbvie.com/our-science/products.html):

Excluding medical aestetics products and eye care products

1. HUMIRA®

(adalimumab) injection, for subcutaneous use

Learn about HUMIRA®

2. RINVOQ™ **oral BCS1** **2019-1** **10s1**

(upadacitinib) extended-release tablets for o use

Learn about RINVOQ™

https://www.accessdata.fda.gov/drugsatfda_docs/nda/2019/211675Orig1s000ChemR.pdf

3. SKYRIZI®

(risankizumab-rzaa) injection, for subcutaneous use

Learn about SKYRIZI®

Neuroscience
Description

•

4. BOTOX®

(onabotulinumtoxinA) for injection, for intramuscular, intradetrusor, or intradermal use

Learn about BOTOX®

-

5. CELEXA® **oral BCS0 1998 90s0**

(citalopram hydrobromide) tablets

Learn about CELEXA®

-

6. DEPAKOTE® **oral BCS2 1983 80s2**

(divalproex sodium) delayed-release tablets, for o use

Learn about DEPAKOTE®

https://www.accessdata.fda.gov/drugsatfda_docs/nda/2002/020782_s000_Depakote_Chemr.pdf

-

7. DEPAKOTE® ER **oral BCS2 2000-2 00s2**

(divalproex sodium) extended-release tablets, for o use

Learn about DEPAKOTE® ER

-

8. DEPAKOTE® SPRINKLE CAPSULES **oral BCS2 1989 80s2**

(divalproex sodium) delayed release capsules, for o use

Learn about DEPAKOTE® SPRINKLE CAPSULES

•

9. DUOPA oral BCS1 1975 70s1

(carbidopa and levodopa) enteral suspension

Learn about DUOPA

https://www.accessdata.fda.gov/drugsatfda_docs/nda/2015/203312Orig1s000ClinPharmR.pdf
https://www.accessdata.fda.gov/drugsatfda_docs/nda/2015/203312Orig1s000ClinPharmR.pdf

•

10. FETZIMA® oral BCS1 2013-1 10s1

(levomilnacipran) extended-release capsules, for o use

Learn about FETZIMA®

https://www.accessdata.fda.gov/drugsatfda_docs/nda/2013/204168orig1s000clinpharmr.pdf

•

11. FIORINAL® oral BCS1 BCS3 1976 70s3

(Butalbital, Aspirin, and Caffeine Capsules, USP), CIII

Learn about FIORINAL®

https://www.accessdata.fda.gov/drugsatfda_docs/nda/2009/040885Orig1s000.pdf

10.1002/jps.23212

•

12. FIORINAL® WITH CODEINE oral BCS1 BCS3 1990 90s3
(butalbital, aspirin, caffeine, and codeine phosphate) capsules, for o use, CIII

Learn about FIORINAL® WITH CODEINE

10.1002/jps.23977

- 13. LEXAPRO® oral BCS0 2002-0 00s0

(escitalopram oxalate) o solution

(escitalopram oxalate) tablets, for o use

Learn about LEXAPRO®

- 14. NAMENDA XR® oral BCS1 2003-1 00s1

(memantine hydrochloride) extended release capsules, for o use

Learn about NAMENDA XR®

https://www.accessdata.fda.gov/drugsatfda_docs/nda/2005/021627s000_Namenda_ClinPharmR.pdf

- 15. NAMENDA® oral BCS1 2003-1 00s1

(memantine HCl) tablets, for o use

Learn about NAMENDA®

- 16. NAMZARIC® oral BCS1 2014-1 10s1

(memantine and donepezil hydrochlorides) extended-release capsules, for o use

Learn about NAMZARIC®

https://www.ema.europa.eu/en/documents/assessment-report/balaxur-epar-refusal-public-assessment-report_en.pdf

- **17.** QULIPTA™ oral BCS2 BCS4 2021-4 20s4

(atogepant) tablets, for o use

Learn about QULIPTA™

https://www.accessdata.fda.gov/drugsatfda_docs/label/2021/215206Orig1s000lbl.pdf

-

18. SAPHRIS®

(asenapine) sublingual tablets

Learn about SAPHRIS®

-

19. SAVELLA® **oral BCS1 2009-1 00s1**

(milnacipran HCl) tablets

Learn about SAVELLA®

https://www.accessdata.fda.gov/drugsatfda_docs/label/2016/022256s022lbl.pdf

-

20. UBRELVY™ **oral BCS4 2019-4 10s4**

(ubrogepant) tablets, for o use

Learn about UBRELVY™

https://www.accessdata.fda.gov/drugsatfda_docs/nda/2019/211765Orig1s000ChemR.pdf

-

21. VIIBRYD® **oral BCS0 2011-0 10s0**

(vilazodone hydrochloride) tablets, for o use

Learn about VIIBRYD®

https://www.accessdata.fda.gov/drugsatfda_docs/nda/2011/022567Orig1s000ClinPharmR.pdf

- 22. VRAYLAR® oral BCS2 2015-2 10s2

(cariprazine) capsules, for o use

Learn about VRAYLAR®

https://www.ema.europa.eu/en/documents/assessment-report/reagila-epar-public-assessment-report_en.pdf

Oncology

- 23. VENCLEXTA® oral BCS4 2016-4 10s4

(venetoclax tablets), for o use

Learn about VENCLEXTA®

https://www.ema.europa.eu/en/documents/assessment-report/venclyxto-epar-public-assessment-report_en.pdf

Virology

- 24. KALETRA® oral BCS4 2000-4 00s4

(lopinavir/ritonavir) tablets, for o use and o solution

Learn about KALETRA®

https://www.accessdata.fda.gov/drugsatfda_docs/nda/2010/022417s000_ChemR.pdf

https://www.accessdata.fda.gov/drugsatfda_docs/nda/2010/022417s000_ChemR.pdf

- 25. MAVYRET® oral BCS4 2017-4 10s4

(glecaprevir/pibrentasvir) tablets, for o use

Learn about MAVYRET®

https://www.ema.europa.eu/en/documents/assessment-report/maviret-epar-public-assessment-report_en.pdf

- 26. NORVIR® oral BCS4 1996 90s4

  (ritonavir) tablets, for o use and o solution and oral powder

  Learn about NORVIR®

  https://www.accessdata.fda.gov/drugsatfda_docs/nda/2010/022417s000_ChemR.pdf

- 27. VIEKIRA PAK® oral BCS4 2014-4 10s4

  (ombitasvir, paritaprevir and ritonavir tablets; dasabuvir tablets), co-packaged for o use

  Learn about VIEKIRA PAK®

  https://www.accessdata.fda.gov/drugsatfda_docs/nda/2010/022417s000_ChemR.pdf

Other Specialty Areas

- 28. ACTONEL® oral BCS3 1998 90s3

  (risedronate sodium) tablets, for o use

  Learn about ACTONEL®

  https://www.accessdata.fda.gov/drugsatfda_docs/nda/98/20835_actonel_biopharmr.pdf

- 29. AEROCHAMBER PLUS® FLOW-VU®

  Anti-Static Valved Holding Chamber Small Mask/Medium Mask

  Anti-Static Valved Holding Chamber Mouthpiece/Large Mask

  Learn about AEROCHAMBER PLUS® FLOW-VU®

- 30. ALORA®

(estradiol transdermal system)

Learn about ALORA®

-

31. ANDRODERM®

(testosterone transdermal system), for topical use CIII

Learn about ANDRODERM®

-

32. ANDROGEL®

(testosterone gel) 1.62%

Learn about ANDROGEL®

-

33. AQUADEKS®

chewable tablets (aqua-dex') multivitamin and mineral supplement

Learn about AQUADEKS®

-

34. ARMOUR® THYROID **oral BCS0**

(thyroid tablets, USP)

Learn about ARMOUR® THYROID

-

35. ATELVIA® **oral BCS3 1998 90s3**

(risedronate sodium) delayed-release tablets

Learn about ATELVIA®

https://www.accessdata.fda.gov/drugsatfda_docs/nda/98/20835_actonel_biopharmr.pdf

-

36. AVAGE®

(tazarotene) cream 0.1%, for topical use

Learn about AVAGE®

-

37. AVYCAZ®

(ceftazidime and avibactam) for injection, for intravenous use

Learn about AVYCAZ®

-

38. BYSTOLIC® **oral BCS0 2007-0 00s0**

(nebivolol) tablets, for o use

Learn about BYSTOLIC®

-

39. CONDYLOX® Gel 0.5%

(podofilox gel)

Learn about CONDYLOX® Gel 0.5%

•

40. CREON® **oral BCS0 2009-0 00s0**

(pancrelipase) Delayed-Release Capsules for o use

Learn about CREON®

•

41. CRINONE®

(progesterone gel), for vaginal use

Learn about CRINONE®

•

42. DALVANCE®

(dalbavancin) for injection, for intravenous use

Learn about DALVANCE®

•

43. ENABLEX® **oral BCS0 2004-0 00s0**

(darifenacin) extended-release tablets

Learn about ENABLEX®

•

44. ESTRACE® Cream

(estradiol vaginal cream, USP, 0.01%)

Learn about ESTRACE® Cream

- 45. ESTROSTEP® FE **oral BCS0 1973 70s0**

(norethindrone acetate and ethinyl estradiol tablets, USP and ferrous fumarate tablets)

Learn about ESTROSTEP® FE

- 46. FEMHRT® **oral BCS0 1973 70s0**

(norethindrone acetate/ethinyl estradiol tablets)

Learn about FEMHRT®

- 47. GELNIQUE®

(oxybutynin chloride) 10% gel

Learn about GELNIQUE®

- 48. GENERESS® FE

(norethindrone and ethinyl estradiol chewable tablets and ferrous fumarate chewable tablets)

Learn about GENERESS® FE

- **49.** GENGRAF® Capsules **oral BCS0 1983 80s0**

(cyclosporine capsules, USP [modified])

Learn about GENGRAF® Capsules

-

50. GENGRAF® Solution **oral BCS0 1983 80s0**

(cyclosporine o solution, USP [modified])

Learn about GENGRAF® O Solution

-

51. INFED®

(iron dextran Injection USP)

Learn about INFED®

-

52. K-TAB®

(potassium chloride) extended-release tablets, for o use

Learn about K-TAB®

-

53. KADIAN® **oral BCS3 1941 40s3**

(morphine sulfate) extended-release capsules, for o use, CII

Learn about KADIAN®

https://www.accessdata.fda.gov/drugsatfda_docs/nda/2011/201517Orig1s000SumR.pdf

-

54. LILETTA®

(levonorgestrel-releasing intrauterine system) SHI

Learn about LILETTA®

- 55. LO LOESTRIN® FE **oral BCS0 1968 60s0**

(norethindrone acetate and ethinyl estradiol tablets, ethinyl estradiol tablets and ferrous fumarate tablets)

Learn about LO LOESTRIN® FE

- 56. LUPANETA PACK®

(leuprolide acetate for depot suspension and norethindrone acetate tablets), co-packaged for intramuscular use and for o use, respectively

Learn about LUPANETA PACK®

- 57. LUPRON DEPOT-PED®

(leuprolide acetate for depot suspension)

Learn about LUPRON DEPOT-PED®

- 58. LUPRON DEPOT® GYN

(leuprolide acetate for depot suspension)

Learn about LUPRON DEPOT® GYN

- 59. LUPRON DEPOT® URO

(leuprolide acetate for depot suspension)

Learn about LUPRON DEPOT® URO

- 60. MINASTRIN® 24 Fe **oral BCS0 1968 60s0**

(norethindrone acetate and ethinyl estradiol tablets and ferrous fumarate tablets)

Learn about MINASTRIN® 24 Fe

- 61. MONUROL® **oral BCS1 1996 90s1**

(fosfomycin tromethamine) granules for o solution

Learn about MONUROL®

https://www.accessdata.fda.gov/drugsatfda_docs/nda/96/050717Orig1s000rev.pdf

- 62. NEPHRO-VITE®RX

(vitamin B complex and C supplement)

Learn about NEPHRO-VITE®RX

- 63. NIASPAN® oral BCS1 1997 90s1

(niacin extended-release tablets) for o use

Learn about NIASPAN®

https://www.accessdata.fda.gov/drugsatfda_docs/nda/2008/022078s000_ChemR.pdf

- 64. NIMBEX®

(cisatracurium besylate) injection, for intravenous use

Learn about NIMBEX®

- 65. NORCO® **oral BCS1 1982 80s2**

(hydrocodone bitartrate and acetaminophen tablets, USP) CII

Learn about NORCO®

https://www.accessdata.fda.gov/drugsatfda_docs/nda/2014/205474Orig1s000ClinPharmR.pdf

https://www.ema.europa.eu/en/documents/scientific-guideline/draft-paracetamol-oral-use-immediate-release-formulations-product-specific-bioequivalence-guidance_en.pdf

- 66. ORIAHNN® **oral BCS0 2020-0 20s0**

(elagolix, estradiol, and norethindrone acetate capsules; elagolix capsules) co-packaged for o use

Learn about ORIAHNN®

- 67. ORILISSA® **oral BCS3 2018-3 10s3**

(elagolix) tablets, for o use

Learn about ORILISSA®

https://www.accessdata.fda.gov/drugsatfda_docs/nda/2018/210450Orig1s000ChemR.pdf

•

68. OXYTROL®

(oxybutynin transdermal system)

Learn about OXYTROL®

•

69. RAPAFLO® **oral BCS4 2008-4 00s4**

(silodosin) capsules, for o use

Learn about RAPAFLO®

https://www.accessdata.fda.gov/drugsatfda_docs/label/2013/022206s012lbl.pdf

•

70. RECTIV®

(nitroglycerin) ointment 0.4%, for intra-anal use

Learn about RECTIV®

•

71. SURVANTA®

(beractant) intratracheal suspension

Learn about SURVANTA®

•

72. SYNTHROID® **oral BCS0 2000-0 00s0**

(levothyroxine sodium) tablets, for o use

Learn about SYNTHROID®

https://www.ema.europa.eu/en/documents/scientific-guideline/levothyroxine-tablets-125-mcg-25-mcg-50-mcg-75-mcg-100-mcg-200-mcg-additional-strengths-within-range_en.pdf

•

73. TARKA® **oral BCS1 1996 90s1**

(trandolapril/verapamil HCI ER tablets)

Learn about TARKA®

https://www.accessdata.fda.gov/drugsatfda_docs/label/2016/018925s010lbl.pdf

https://www.accessdata.fda.gov/drugsatfda_docs/label/2012/020528s020,020591s020lbl.pdf

•

74. TAYTULLA® **oral BCS0 1968 60s0**

(norethindrone acetate and ethinyl estradiol capsules and ferrous fumarate capsules), for o use

Learn about TAYTULLA®

•

75. TEFLARO®

(ceftaroline fosamil) for injection, for intravenous use

Learn about TEFLARO®

•

76. THYROLAR® Tablets **oral BCS0**

(liotrix tablets, USP)

Learn about THYROLAR® Tablets

•

77. TRICOR® **oral BCS2 1993 90s2**

(fenofibrate) tablets, for o use

Learn about TRICOR®

https://www.accessdata.fda.gov/drugsatfda_docs/nda/2007/022118s000_ChemR.pdf

•

78. TRILIPIX® **oral BCS2 2008-2 00s2**

(fenofibric acid) delayed-release capsules

Learn about TRILIPIX®

https://www.accessdata.fda.gov/drugsatfda_docs/nda/2007/022118s000_ChemR.pdf

•

79. ULTANE®

(sevoflurane) volatile liquid for inhalation

Learn about ULTANE®

•

80. VANIQA®

(eflornithine hydrochloride) cream, 13.9%

Learn about VANIQA®

•

81. ZEMPLAR® **oral BCS0 1998 90s0**

(paricalcitol) injection

(paricalcitol) capsules, for o use

Gastroenterology
Description

- **82.** ACTIGALL® **oral BCS2 BCS4 1987 80s4**

(ursodiol, USP) capsules

Learn about ACTIGALL®

https://www.ema.europa.eu/en/documents/scientific-guideline/ursodeoxycholic-acid-capsule-250-mg-film-coated-tablet-150-mg-300-mg-450-mg-500-mg-600-mg-suspension/ml-250-mg/5-ml-product-specific-bioequivalence-guidance_en.pdf

- 83. ASACOL® HD **oral BCS0 1987 80s0**

(mesalamine) delayed-release tablets, for o use

Learn about ASACOL® HD

- 84. BENTYL®

(dicyclomine hydrochloride) injection, for intramuscular use

Learn about BENTYL®

- 85. CANASA®

(mesalamine) rectal suppository

Learn about CANASA®

- 86. CARAFATE® **oral BCS0 1981 80s0**

(sucralfate) tablets

(sucralfate) suspension

Learn about CARAFATE®

- 87. DELZICOL® **oral BCS4** **1987** **80s4**

(mesalamine) delayed-release capsules, for o use

Learn about DELZICOL®

- 88. LINZESS® **oral BCS3** **2012-3** **10s3**

(linaclotide) capsules, for o use

Learn about LINZESS®

https://www.accessdata.fda.gov/drugsatfda_docs/nda/2012/202811Orig1s000ClinPharmR.pdf

- 89. PYLERA® **oral BCS0** **2006-0** **00s0**

(bismuth subcitrate potassium, metronidazole, tetracycline hydrochloride) capsules, for o use

Learn about PYLERA®

- 90. URSO 250® and URSO FORTE® **oral BCS2** **1987** **80s2**

(ursodiol) tablets, for o use

Learn about URSO 250® and URSO FORTE®

•

91. VIBERZI® oral BCS3 2015-3 10s3

(eluxadoline) tablets, for o use, CIV

Learn about VIBERZI®

https://www.accessdata.fda.gov/drugsatfda_docs/nda/2015/206940Orig1s000ClinPharmR.pdf

GlaxoSmithKline products:

GlaxoSmithKline products available worldwide
https://www.gsk.com/en-gb/products/products-a-z/

ANALYSIS EXCLUDES GSK PRODUCTS THAT ARE VACCINES!

Ambirix hepatitis A (inactivated) and hepatitis B (rDNA) (HAB) vaccine (adsorbed) Vaccines

1. Amoxil amoxiicillin General medicines
   Oral BCS1 10.1016/j.xphs.2017.04.068 1974 70s1
2. Anoro Ellipta umeclidinium and vilanterol General medicines
3. Apretude cabotegravir Specialty medicines
4. Arnuity Ellipta Fluticasone General medicines
5. Augmentin amoxicillin/clavulanate potassium General medicines
   Oral BCS0 1984 80s0
6. Avamys / Veramyst fluticasone furoate General medicines
7. Avodart/Avolve / Duodart dutasteride General medicines
   Oral BCS4 2001-4 00s4
   https://www.accessdata.fda.gov/drugsatfda_docs/label/2008/021319s015lbl.pdf
8. Bactroban mupirocin General medicines
9. Beconase beclomethasone General medicines
10. Becotide/Becloforte beclomethasone dipropionate General medicines
11. Benlysta belimumab Specialty medicines
12. Betnovate betamethasone valerate General medicines

Bexsero meningitis B Vaccines

13. Blenrep belantamab mafodotin Specialty medicines

Boostrix adsorbed tetanus toxoid, reduced diphtheria toxoid and acellular pertussis vaccine, Vaccines

Boostrix IPV Diphtheria, tetanus, pertussis (acellular, component) and poliomyelitis (inactivated) vaccine (adsorbed, reduced antigen(s) content) Vaccines

14. Cabenuva cabotegravir/rilpivirine Specialty medicines

15. Cabenuva/Vocabria + Rekambys Cabotegravir, rilpivirine Specialty medicines

Cervarix human Papillomavirus vaccine (types 16, 18) - recombinant, adjuvanted, adsorbed Vaccines

16. Combivir lamivudine/ zidovudine Specialty medicines
 Oral BCS3 1997 90s3
 https://www.ema.europa.eu/en/documents/assessment-report/lamivudine/zidovudine-teva-epar-public-assessment-report_en.pdf
17. Cutivate fluticasone propionate General medicines
18. Dermovate clobetasol propionate General medicines
19. Dovato dolutegravir/lamivudine Specialty medicines
 Oral BCS2 2019-2 10s2
 https://www.ema.europa.eu/en/documents/scientific-guideline/draft-dolutegravir-film-coated-tablets-10-mg-25-mg-50-mg-product-specific-bioequivalence-guidance_en.pdf
 https://www.accessdata.fda.gov/drugsatfda_docs/nda/2019/211994Orig1s000ChemR.pdf
20. Duac clindamycin phosphate and benzoyl peroxide General medicines
21. Eltroxin Levothyroxin sodium General medicines
 Oral BCS0
 https://www.ema.europa.eu/en/documents/scientific-guideline/levothyroxine-tablets-125-mcg-25-mcg-50-mcg-75-mcg-100-mcg-200-mcg-additional-strengths-within-range_en.pdf

Engerix hepatitis B Vaccine (Recombinant) Vaccines

22. Epivir lamivudine Specialty medicines
 Oral BCS3 1995 90s3
23. Epzicom/ Kivexa abacavir/ lamivudine Specialty medicines
 Oral BCS3 2004-3 00s3
 https://extranet.who.int/pqweb/sites/default/files/HA662Part6v02.pdf
24. Eumovate clobetasone butyrate General medicines

Fendrix hepatitis B (rDNA) vaccine (adjuvanted, adsorbed) Vaccines

25. Flixonase/Flonase fluticasone propionate General medicines
26. Flixotide / Flovent - Diskus / MDI fluticasone propionate General medicines
27. Flolan epoprostenol General medicines

Fluarix QIV seasonal influenza vaccine (4 strain) Vaccines

Flulaval QIV seasonal influenza vaccine (4 strain) Vaccines

28. Fortum ceftazidime General medicines

Havrix hepatitis A vaccine, inactivated Vaccines

29. Hepsera Adefovir General medicines
 Oral BCS3 2002-3 00s3
 https://www.accessdata.fda.gov/drugsatfda_docs/pediatric/021449S011_Adefovir_Clinpharm_Review_BPCA.pdf

Hiberix haemophilus B conjugate vaccine (tetanus toxoid conjugate) Vaccines

30. Imigran/ImITrex FDT sumatriptan succinate General medicines
 Oral BCS3 1992 90s3
 https://www.ema.europa.eu/en/documents/assessment-report/sumatriptan-galpharm-epar-public-assessment-report_en.pdf
31. Imuran Azathioprine General medicines

Oral BCS4 1968 60s4
https://www.ema.europa.eu/en/documents/assessment-report/jayempi-h-c-005055-0000-epar-assessment-report_en.pdf

32. Incruse umeclidinium General medicines

Infanrix diphtheria and tetanus toxoids and acellular pertussis vaccine adsorbed Vaccines

Infanrix IPV diphtheria, tetanus, pertussis (acellular, component) and poliomyelitis (inactivated) vaccine (adsorbed) Vaccines

33. Jemperli dostarlimab Specialty medicines
34. Juluca dolutegravir/rilpivirine Specialty medicines
    Oral BCS2 BCS4 2017-4 10s4
    https://www.ema.europa.eu/en/documents/scientific-guideline/rilpivirine-film-coated-tablets-25-mg-product-specific-bioequivalence-guidance-first-version_en.pdf
    https://www.ema.europa.eu/en/documents/scientific-guideline/draft-dolutegravir-film-coated-tablets-10-mg-25-mg-50-mg-product-specific-bioequivalence-guidance_en.pdf
35. Keppra Levetiracetam General medicines
    Oral BCS1 1999 90s1
    https://www.fda.gov/media/89867/download

Kinrix diphtheria and tetanus toxoids and acellular pertussis adsorbed and inactivated poliovirus vaccine Vaccines

36. Lacipil Lacidipine General medicines
    Oral BCS0
37. Lacticare - Family Lactic Acid General medicines
38. Lamictal lamotrigine General medicines
    Oral BCS2 1994 90s2
    https://www.accessdata.fda.gov/drugsatfda_docs/nda/2009/022251s000_ChemR.pdf
39. Lanoxin Digoxin General medicines
    Oral BCS4 1954 50s4
    10.1021/acs.molpharmaceut.5b00278
40. Levoxacin Levofloxacin General medicines
    Oral BCS1 1996 90s1
    https://extranet.who.int/pqweb/sites/default/files/documents/BE_levofloxacin_July2021.pdf
41. Lexiva/ Telzir fosamprenavir Specialty medicines
    Oral BCS0 2003-0 00s0
42. Lovaza omega-3 acid ethyl esters General medicines
    Oral BCS0 2004-0 00s0
43. Malarone atovaquone and proguanil hydrochloride General medicines
    Oral BCS2 BCS4 2000-4 00s4
    https://www.accessdata.fda.gov/drugsatfda_docs/label/2008/020500s010lbl.pdf

Menitorix combined Haemophilus influenzae type b and Neisseria meningitidis group C (Hib-MenC) conjugate vaccines Vaccines

Menjugate meningitis C Vaccines

Menveo meningitis ACWY Vaccines

44. Mepron atovaquone General medicines
    Oral BCS2 BCS4 1992 90s4
    https://www.accessdata.fda.gov/drugsatfda_docs/label/2008/020500s010lbl.pdf

45. Naramig/Amerge naratriptan hydrochloride General medicines
Oral BCS1 1998 90s1
https://www.accessdata.fda.gov/drugsatfda_docs/label/2012/020763s008s009lbl.pdf
46. Nucala mepolizumab Specialty medicines
47. Paxil / Seroxat paroxetine hydrochloride General medicines
Oral BCS1 1991 90s1
https://www.accessdata.fda.gov/drugsatfda_docs/nda/2013/204516Orig1s000ClinPharmR.pdf

Pediarix diphtheria and tetanus toxoids and acellular pertussis adsorbed, hepatitis B (recombinant) and inactivated poliovirus vaccine combined Vaccines
Priorix measles, mumps and rubella vaccine (live attenuated virus) Vaccines
Priorix-Tetra measles, mumps, rubella, varicella (chicken pox) Vaccines

48. Relenza zanamivir General medicines
49. Relvar/Breo Fluticasone furoate/vilanterol General medicines
50. Requip ropinirole hydrochloride General medicines
Oral BCS1 1997 90s1
https://www.accessdata.fda.gov/drugsatfda_docs/label/2008/020658s018s020s021lbl.pdf

51. Retrovir zidovudine Specialty medicines
Oral BCS3 1987 80s3
https://www.ema.europa.eu/en/documents/assessment-report/lamivudine/zidovudine-teva-epar-public-assessment-report_en.pdf

Rotarix human rotavirus vaccine, live attenuated Vaccines

52. Rukobia fostemsavir Specialty medicines
Oral BCS0 2020-0 20s0
53. Rythmol propafenone hydrochloride General medicines
Oral BCS0
54. Selzentry/ Celsentri maraviroc Specialty medicines
Oral BCS3 2007-3 00s3
https://www.ema.europa.eu/en/documents/variation-report/celsentri-h-c-000811-x-0046-g-epar-assessment-report-variation_en.pdf
55. Septrin Cotrimoxazole General medicines
Oral BCS0
56. Seretide / Advair - Diskus / MDI salmeterol xinofoate, fluticasone propionate General medicines
57. Serevent - Diskus / MDI salmeterol xinafoate General medicines

Shingrix Zoster Vaccine Recombinant, Adjuvanted Vaccines
Synflorix pneumococcal polysaccharide conjugate vaccine (adsorbed) Vaccines

58. Tivicay dolutegravir Specialty medicines
Oral BCS2 BCS4 2013-4 10s4
https://www.ema.europa.eu/en/documents/scientific-guideline/draft-dolutegravir-film-coated-tablets-10-mg-25-mg-50-mg-product-specific-bioequivalence-guidance_en.pdf
59. Toctino Alitretinoin General medicines
Oral BCS0 1999 90s0

60. Trelegy - Ellipta fluticasone furoate, umeclidinium, and vilanterol inhalation powder General medicines
61. Triumeq dolutegravir/ abacavir/ lamivudine Specialty medicines
    Oral BCS2 BCS4 2014-4 10s4
    https://www.ema.europa.eu/en/documents/scientific-guideline/draft-dolutegravir-film-coated-tablets-10-mg-25-mg-50-mg-product-specific-bioequivalence-guidance_en.pdf
62. Trizivir abacavir/ lamivudine/ zidovudine Specialty medicines
    Oral BCS3 2000-3 00s3
    https://extranet.who.int/pqweb/sites/default/files/HA662Part6v02.pdf
    https://www.ema.europa.eu/en/documents/assessment-report/lamivudine/zidovudine-teva-epar-public-assessment-report_en.pdf

Twinrix combined hepatitis A (inactivated virus) and hepatitis B vaccine (genetically derived surface antigen) Vaccines

63. Valtrex valaciclovir hydrochloride General medicines
    Oral BCS3 1995 90s3
    https://www.fda.gov/media/71296/download

Varilrix varicella (chicken pox) Vaccines

64. Ventolin - Diskus / MDI Salbutamol General medicines
65. Viracept nelfinavir Specialty medicines
    Oral BCS0 1997 90s0
66. Vocabria cabotegravir Specialty medicines
    Oral BCS2 2021-2 20s2
    https://www.ema.europa.eu/en/documents/assessment-report/vocabria-epar-public-assessment-report_en.pdf
67. Volibris (Ambrisentan) ambrisentan General medicines
    Oral BCS2 2007-2 00s2
    https://www.ema.europa.eu/en/documents/assessment-report/ambrisentan-mylan-epar-public-assessment-report_en.pdf
68. Wellbutrin bupropion hydrochloride General medicines
    Oral BCS1 1985 80s1
    https://www.accessdata.fda.gov/drugsatfda_docs/nda/2013/020358Orig1s053.pdf
69. Xevudy sotrovimab Specialty medicines
70. Xyzal Levocetirizine General medicines
    Oral BCS3 1995 90s3
    https://www.ema.europa.eu/en/documents/referral/gvk-biosciences-article-31-referral-gvk-biosciences-final-assessment-report-following-re-examination_en.pdf
71. Zagallo Dutasteride General medicines
    Oral BCS4 2001-4 00s4
    https://www.accessdata.fda.gov/drugsatfda_docs/label/2008/021319s015lbl.pdf
72. Zeffix lamivudine General medicines
    Oral BCS3 1995 90s3
    https://www.ema.europa.eu/en/documents/assessment-report/lamivudine/zidovudine-teva-epar-public-assessment-report_en.pdf
73. Zejula niraparib Specialty medicines
    Oral BCS2 2017-2 10s2

https://www.ema.europa.eu/en/documents/assessment-report/zejula-epar-public-assessment-report_en.pdf

74. Zentel Albendazole General medicines
Oral BCS2 BCS4 1996 90s4
https://www.accessdata.fda.gov/drugsatfda_docs/label/2009/020666s005s006lbl.pdf
75. Ziagen abacavir Specialty medicines
Oral BCS3 1998 90s3
https://extranet.who.int/pqweb/sites/default/files/HA662Part6v02.pdf
76. Zinacef cefuroxime General medicines
Oral BCS0 1983 80s0
77. Zovirax aciclovir General medicines
78. Zyban bupropion hydrochloride General medicines
Oral BCS1 1985 80s1
https://www.accessdata.fda.gov/drugsatfda_docs/nda/2013/020358Orig1s053.pdf
79. Zyloric Allopurinol General medicines
Oral BCS4 1966 60s4
https://www.ema.europa.eu/en/documents/assessment-report/duzallo-epar-public-assessment-report_en.pdf
80. Zyrtec cetirizine General medicines
Oral BCS3 1995 90s3
https://www.ema.europa.eu/en/documents/referral/gvk-biosciences-article-31-referral-gvk-biosciences-final-assessment-report-following-re-examination_en.pdf

Johnson & Johnson (Janssen) products:

Janssen products (US market)
https://www.janssen.com/us/our-products

1. BALVERSA™ erdafitinib **oral BCS1 2019-1 20s1**
https://www.accessdata.fda.gov/drugsatfda_docs/nda/2019/212018Orig1s000ChemR.pdf
2. CARVYKTI™ ciltacabtagene autoleucel; cilta-cel
3. CONCERTA® methylphenidate HCl **oral BCS1 1955 50s1**
https://www.ema.europa.eu/en/documents/referral/methylphenidate-hexal-article-29-referral-assessment-report_en.pdf
4. DARZALEX FASPRO™ daratumumab and hyaluronidase-fihj
5. DARZALEX® daratumumab
6. DITROPAN XL® oxybutynin chloride **oral BCS0 1975 70s0**
7. EDURANT® rilpivirine **oral BCS2 BCS4 2011-4 10s4**
8. ELMIRON® pentosan polysulfate sodium **oral BCS3 1996 90s3**
https://www.ema.europa.eu/en/documents/assessment-report/elmiron-epar-public-assessment-report_en.pdf

9. ERLEADA™ apalutamide 60mg tablets **oral BCS2 2018-2 10s2**
https://www.accessdata.fda.gov/drugsatfda_docs/nda/2018/210951Orig1s000ChemR.pdf
10. HALDOL® haloperidol
11. HALDOL® Decanoate
(haloperidol decanoate)
12. IMBRUVICA® ibrutinib **oral BCS2 2013-2 10s2**
https://www.accessdata.fda.gov/drugsatfda_docs/nda/2018/210563Orig1s000,210563Orig2s000ChemR.pdf
13. INFLIXIMAB
14. INTELENCE® etravirine **oral BCS4 2008-4 00s4**
https://www.ema.europa.eu/en/documents/assessment-report/intelence-epar-public-assessment-report_en.pdf
15. INVEGA HAFYERA™ paliperidone palmitate
16. INVEGA SUSTENNA® paliperidone palmitate
17. INVEGA TRINZA® paliperidone palmitate
18. INVEGA® paliperidone **oral BCS4 2006-4 00s4**
https://www.accessdata.fda.gov/drugsatfda_docs/label/2010/021999s018lbl.pdf
19. INVOKAMET® XR canagliflozin and metformin HCl extended-release **oral BCS4**
2014-4 10s4
https://www.accessdata.fda.gov/drugsatfda_docs/nda/2014/204353Orig1s000ClinPharmR.pd
20. INVOKAMET® canagliflozin and metformin HCl **oral BCS4**
2014-4 10s4
https://www.accessdata.fda.gov/drugsatfda_docs/nda/2014/204353Orig1s000ClinPharmR.pdf
21. INVOKANA® canagliflozin **oral BCS4**
2013-4 10s4
https://www.accessdata.fda.gov/drugsatfda_docs/nda/2014/204353Orig1s000ClinPharmR.pdf
22. OPSUMIT® macitentan **oral BCS2**
2013-2 10s2
https://www.accessdata.fda.gov/drugsatfda_docs/nda/2013/204410Orig1s000ClinPharmR.pdf
23. PONVORY™ Ponesimod **oral BCS4**
2021-4 20s4
https://www.accessdata.fda.gov/drugsatfda_docs/nda/2021/213498Orig1s000ChemR.pdf
24. PREZCOBIX® darunavir 800 mg/ cobicistat 150 mg **oral BCS2**
2015-2 10s2
https://www.ema.europa.eu/en/documents/assessment-report/darunavir-krka-epar-public-assessment-report_en.pdf
https://www.ema.europa.eu/en/documents/scientific-guideline/draft-elvitegravir/cobicistat/emtricitabine/tenofovir-disoproxil-film-coated-tablets-150-mg/150-mg/200-mg/245-mg-product-specific-bioequivalence-guidance_en.pdf

25. PREZISTA® darunavir oral BCS2
2006-2 00s2
https://www.ema.europa.eu/en/documents/assessment-report/darunavir-krka-epar-public-assessment-report_en.pdf
26. PROCRIT® Epoetin alfa
27. RAZADYNE® ER galantamine HBrq oral BCS0
2001-0 00s0
28. REMICADE® infliximab
29. RISPERDAL CONSTA® risperidone
30. RISPERDAL® Oral Solution risperidone oral BCS2
1993 90s2
31. RISPERDAL® Tablets risperidone oral BCS2
1993 90s2
https://www.accessdata.fda.gov/drugsatfda_docs/label/2005/020272s042,020588s030,021444s016,021346s010lbl.pdf
32. RYBREVANT™ amivantamab-vmjw)
33. SIMPONI ARIA®
34. SIMPONI® golimumab
35. SIRTURO® Tablets bedaquiline oral BCS2
2012-2 10s2
https://www.accessdata.fda.gov/drugsatfda_docs/nda/2012/204384Orig1s000ClinPharmR.pdf
36. SPORANOX® Capsules itraconazole oral BCS2
1992 90s2
https://www.accessdata.fda.gov/drugsatfda_docs/nda/2010/022484Orig1s000ClinPharmR.pdf
37. SPORANOX® Oral Solution itraconazole oral BCS2
1992 90s2
https://www.accessdata.fda.gov/drugsatfda_docs/nda/2010/022484Orig1s000ClinPharmR.pdf
38. SPRAVATO® esketamine nasal spray, CIII
39. STELARA® ustekinumab
40. SYMTUZA® (darunavir/cobicistat/emtricitabine/tenofovir alafenamide tablets 800mg/150mg/200mg/10mg) oral BCS2
2018-2 10s2
41. TECVAYLI™ teclistamab-cqyv
42. TOPAMAX® topiramate oral BCS1
1996 90s1
43. TRACLEER® bosentan oral BCS2
2001-2 00s2
https://www.ema.europa.eu/en/documents/variation-report/tracleer-h-c-401-x-0039-epar-assessment-report-extension_en.pdf

44. TREMFYA® guselkumab

45. ULTRACET® tramadol hydrochloride/acetaminophen **oral BCS1 BCS3**
2001-3 00s3
https://www.accessdata.fda.gov/drugsatfda_docs/label/2009/020281s032s033lbl.pdf

46. ULTRAM® tramadol hydrochloride **oral BCS1 BCS3**
1995 90s3
https://www.accessdata.fda.gov/drugsatfda_docs/label/2009/020281s032s033lbl.pdf

47. UPTRAVI® selexipag **oral BCS2 BCS4**
2015-5 10s4
https://www.ema.europa.eu/en/documents/assessment-report/uptravi-epar-public-assessment-report_en.pdf

48. VELETRI® epoprostenol

49. VENTAVIS® iloprost)

50. VERMOX™ CHEWABLE mebendazole chewable tablets **oral BCS0**
1974 70s0

51. XARELTO® rivaroxaban tablets **oral BCS2**
2011-2 10s2
https://www.ema.europa.eu/en/documents/variation-report/xarelto-h-c-944-x-0010-epar-assessment-report-extension_en.pdf

52. YONDELIS® trabectedin

53. ZAVESCA® miglustat **oral BCS0**
2003-0 00s0
https://www.ema.europa.eu/en/documents/scientific-guideline/miglustat-hard-capsules-100-mg-product-specific-bioequivalence-guidance_en.pdf

54. ZYTIGA® abiraterone acetate **oral BCS2 BCS4**
2011-4 10s4
https://www.ema.europa.eu/en/documents/scientific-guideline/draft-abiraterone-tablets-250-mg-500-mg-product-specific-bioequivalence-guidance-revision-1_en.pdf

Sanofi products:

Products on the US market
https://www.sanofi.us/en/products-and-resources/prescription-products

1. **Adlyxin® (lixisenatide injection)**
2. **ADMELOG® (insulin lispro injection) 100 Units/mL**
3. **Aldurazyme® (laronidase)**
4. **Alprolix® [Coagulation Factor IX (Recombinant), Fc Fusion Protein]**
5. **Amaryl® (glimepiride tablets) oral BCS2 BCS4 1995 90s4**
https://www.ema.europa.eu/en/documents/referral/glimepirida-parke-davis-article-294-referral-assessment-report_en.pdf
6. **Ambien® (zolpidem tartrate) tablets oral BCS1 1992 90s1**

https://doi.org/10.1021/acs.molpharmaceut.5b00076

7. **Ambien CR® (zolpidem tartrate) extended-release tablets** oral BCS1 2005-1 00s1
8. **Apidra® (insulin glulisine injection) 100 Units/mL**
9. **Arava® Tablets (leflunomide)** oral BCS2 1998 90s2
   10.1002/jps.20382
10. **AUBAGIO® (teriflunomide)** oral BCS2 2012-2 10s2
    https://www.ema.europa.eu/en/documents/assessment-report/aubagio-epar-public-assessment-report_en.pdf
11. **Avalide® (irbesartan-hydrochlorothiazide)** oral BCS2 BCS4 1997 90s4
    https://www.accessdata.fda.gov/drugsatfda_docs/label/2011/020757s055lbl.pdf
12. **Avapro® (irbesartan)** oral BCS2 BCS4 1997 90s4
13. **Cablivi® (caplacizumab-yhdp)**
14. **Caprelsa® (vandetanib) Tablets** oral BCS2 2011-2 10s2
    https://www.accessdata.fda.gov/drugsatfda_docs/nda/2011/022405Orig1s000ChemR.pdf
15. **Cerdelga® (eliglustat) capsules** oral BCS3 2014-3 10s3
    https://www.ema.europa.eu/en/documents/assessment-report/cerdelga-epar-public-assessment-report_en.pdf
16. **Cerezyme® (imiglucerase) for injection**
17. **Clolar® (clofarabine injection)**
18. **Dupixent® (dupilumab) Injection**
19. **Elitek® (rasburicase)**
20. **Eloctate® [Antihemophilic Factor (Recombinant), Fc Fusion Protein]**
21. **Eloxatin® (oxaliplatin injection)**
22. **ENJAYMO™ (sutimlimab-jome) injection, for intravenous use**
23. **Fabrazyme® (agalsidase beta)**
24. **Ferrlecit® (sodium ferric gluconate complex in sucrose injection)**
25. **Flomax® (tamsulosin HCI)** oral BCS0 1997 90s0
26. **Hectorol® (doxercalciferol)**
27. **Insulin Glargine injection 100 Units/mL (U-100)**
28. **Jevtana® (cabazitaxel) Injection**
29. **Kevzara® (sarilumab) Injection**
30. **Lantus® (insulin glargine injection) 100 Units/mL**
31. **LEMTRADA® (alemtuzumab)**
32. **Lovenox® (enoxaparin sodium injection)**
33. **Lumizyme® (alglucosidase alfa)**
34. **Mozobil® (plerixafor) injection**
35. **Multaq® (dronedarone)** oral BCS2 BCS4 2009-4 00s4
    https://www.ema.europa.eu/en/documents/scientific-guideline/draft-dronedarone-film-coated-tablets-400-mg-product-specific-bioequivalence-guidance-first-version_en.pdf
36. **Nexviazyme™ (avalglucosidase alfa-ngpt)**
37. **Plavix® (clopidogrel bisulfate)** oral BCS2 BCS4 1997 90s4
    https://www.accessdata.fda.gov/drugsatfda_docs/label/2009/020839s044lbl.pdf
38. **Priftin® (rifapentine) Tablets** oral BCS2 1998-2 90s2
    https://www.usp-pqm.org/sites/default/files/pqms/article/rifapentine-pir-jul2018.pdf
39. **Primaquine® (Primaquine Phosphate Tablets)** oral BCS0 1952 50s0
40. **Renagel® Tablets (sevelamer hydrochloride) 800mg Tablets** oral BCS0 2000 00s0
41. **Renvela® (sevelamer carbonate)** oral BCS0 2000-0 00s0
42. **Rezurock® (belumosudil) tablets** oral BCS4
43. **Rifadin® IV (rifampin for injection, USP)**
44. **Sarclisa® (isatuximab-irfc)**
45. **Soliqua® 100/33 (insulin glargine and lixisenatide) injection 100 Units/mL and 33 mcg/mL**
46. **Synvisc-One® (hylan G-F 20)**
47. **Synvisc® (hylan G-F 20)**
48. **Taxotere® (docetaxel) Injection**
49. **Thymoglobulin® [Anti-thymocyte Globulin (Rabbit)]**
50. **Thyrogen® (thyrotropin alfa)**

51. **Toujeo® (insulin glargine injection) 300 Units/mL**
52. **Xenpozyme™ (olipudase alfa-rpcp)**
53. **Zaltrap® (ziv-aflibercept) Injection for Intravenous Infusion**

Merck products:

https://www.merck.com/products/

products intended for use in the US only, excluding vaccines

1. ANTIVENIN™ (LATRODECTUS MACTANS)

BCG Vaccine (For Percutaneous Use)

2. BELSOMRA® (suvorexant) tablets, for o use, C-IV
   Oral BCS2 2014-2 10s2
   https://www.accessdata.fda.gov/drugsatfda_docs/nda/2014/204569Orig1s000ClinPharmR.pdf
3. BRIDION® (sugammadex) Injection, for intravenous use
4. CANCIDAS® (caspofungin acetate) for Injection, for intravenous use
5. CRIXIVAN® (INDINAVIR SULFATE) CAPSULES
   Oral BCS0 1996 90s0
6. CUBICIN® (daptomycin for injection) for Intravenous Use
7. CUBICIN® RF (daptomycin for injection), for intravenous use
8. DELSTRIGO™ (doravirine, lamivudine, and tenofovir disoproxil fumarate) tablets, for o use
   Oral BCS0 2018-0 10s0
9. DIFICID® (fidaxomicin) for O Suspension
   Oral BCS4 2011-4 10s4
   https://www.accessdata.fda.gov/drugsatfda_docs/nda/2011/201699Orig1s000ClinPharmR.pdf
10. DIFICID® (fidaxomicin) tablets, for o use
    Oral BCS4 2011-4 10s4
    https://www.accessdata.fda.gov/drugsatfda_docs/nda/2011/201699Orig1s000ClinPharmR.pdf
11. EMEND® (aprepitant) capsules (aprepitant) for ol suspension
    Oral BCS2 BCS4 2003-4 00s4
    https://www.ema.europa.eu/en/documents/scientific-discussion/emend-epar-scientific-discussion_en.pdf
12. EMEND® (fosaprepitant) for injection, for intravenous use
13. ENTEREG® (alvimopan) capsules, for o use
    Oral BCS2 BCS4 2008-4 00s4
    https://www.accessdata.fda.gov/drugsatfda_docs/label/2008/021775lbl.pdf

ERVEBO® (Ebola Zaire Vaccine, Live) Suspension for intramuscular injection

14. FOLLISTIM® AQ Cartridge (follitropin beta injection) for subcutaneous use

GARDASIL® [Human Papillomavirus Quadrivalent (Types 6, 11, 16 and 18) Vaccine, Recombinant]

GARDASIL®9 (Human Papillomavirus 9-valent Vaccine, Recombinant) Suspension for intramuscular injection

15. INTRON® A (interferon alfa-2b, recombinant For Injection)
16. INVANZ® (ertapenem for injection) for intravenous (IV) or intramuscular (IM) use
17. ISENTRESS® (raltegravir) chewable tablets, for o use
    Oral BCS2 2007-2 00s2

https://www.ema.europa.eu/en/documents/scientific-discussion/isentress-epar-scientific-discussion_en.pdf

18. ISENTRESS® (raltegravir) film-coated tablets, for o use
    Oral BCS2 2007-2 00s2
    https://www.ema.europa.eu/en/documents/scientific-discussion/isentress-epar-scientific-discussion_en.pdf
19. ISENTRESS® (raltegravir) for o suspension
    Oral BCS2 2007-2 00s2
    https://www.ema.europa.eu/en/documents/scientific-discussion/isentress-epar-scientific-discussion_en.pdf
20. ISENTRESS® HD (raltegravir) film-coated tablets, for o use
    Oral BCS2 2007-2 00s2
    https://www.ema.europa.eu/en/documents/scientific-discussion/isentress-epar-scientific-discussion_en.pdf
21. JANUMET® (sitagliptin and metformin hydrochloride) tablets, for o use
    Oral BCS3 2007-3 00s3
    doi.org/10.1016/j.xphs.2021.01.011
    https://www.ema.europa.eu/en/documents/scientific-guideline/sitagliptin-film-coated-tablets-25-50-100-mg-product-specific-bioequivalence-guidance_en.pdf
22. JANUMET® XR (sitagliptin and metformin hydrochloride extended-release) tablets, for o use
    Oral BCS3 2012-3 10s3
    doi.org/10.1016/j.xphs.2021.01.011
    https://www.ema.europa.eu/en/documents/scientific-guideline/sitagliptin-film-coated-tablets-25-50-100-mg-product-specific-bioequivalence-guidance_en.pdf
23. JANUVIA® (sitagliptin) tablets, for o use
    Oral BCS1 2006-1 00s1
    https://www.ema.europa.eu/en/documents/scientific-guideline/sitagliptin-film-coated-tablets-25-50-100-mg-product-specific-bioequivalence-guidance_en.pdf
24. KEYTRUDA® (pembrolizumab) injection, for intravenous use

M-M-R® II [MEASLES, MUMPS, and RUBELLA VIRUS VACCINE LIVE]

25. NOXAFIL® (posaconazole) injection, for intravenous use, delayed-release tablets, for o use, and o suspension
    Oral BCS2 2006-2 00s2
    https://www.ema.europa.eu/en/documents/scientific-guideline/draft-posaconazole-product-specific-bioequivalence-guidance_en.pdf

Liquid Pedvax HIB® [Haemophilus b Conjugate Vaccine (Meningococcal Protein Conjugate)]

26. PIFELTRO™ (doravirine) tablets, for o use
    Oral BCS2 2018-2 10s2
    https://www.accessdata.fda.gov/drugsatfda_docs/nda/2018/210806Orig1s000,%20210807Orig1s000ChemR.pdf

PNEUMOVAX® 23 [pneumococcal vaccine polyvalent] Sterile, Liquid Vaccine for Intramuscular or Subcutaneous Injection

27. Pregnyl® (chorionic gonadotropin for injection, USP)
28. PREVYMIS™ (letermovir) injection, for intravenous use
29. PREVYMIS™ (letermovir) tablets, for o use
    Oral BCS2 2017-2 10s2

https://www.ema.europa.eu/en/documents/assessment-report/prevymis-epar-public-assessment-report_en.pdf

30. PRIMAXIN® I.V. (imipenem and cilastatin) for Injection, for intravenous use

ProQuad® [Measles, Mumps, Rubella and Varicella Virus Vaccine Live] Lyophilized preparation for subcutaneous injection

31. PROVENTIL® HFA (albuterol sulfate) Inhalation Aerosol
32. RECARBRIO™ (imipenem, cilastatin, and relebactam) for injection, for intravenous use

RECOMBIVAX HB® [HEPATITIS B VACCINE (RECOMBINANT)]

RotaTeq® [Rotavirus Vaccine, Live, O, Pentavalent] O Solution

33. SEGLUROMET™ (ertugliflozin and metformin) tablets, for o use
    Oral BCS3 2017-3 10s3
    https://www.ema.europa.eu/en/documents/assessment-report/steglatro-epar-public-assessment-report_en.pdf
    doi.org/10.1016/j.xphs.2021.01.011
34. SIVEXTRO® (tedizolid phosphate) for injection, for intravenous use
35. SIVEXTRO® (tedizolid phosphate) tablet, for o use
    Oral BCS0 2014-0 10s0
36. STEGLATRO™ (ertugliflozin) tablets, for o use
    Oral BCS1 2017-1 10s1
    https://www.ema.europa.eu/en/documents/assessment-report/steglatro-epar-public-assessment-report_en.pdf
37. STEGLUJAN™ (ertugliflozin and sitagliptin) tablets, for o use
    Oral BCS1 2017-1 10s1
    https://www.ema.europa.eu/en/documents/assessment-report/steglatro-epar-public-assessment-report_en.pdf
    https://www.ema.europa.eu/en/documents/scientific-guideline/sitagliptin-film-coated-tablets-25-50-100-mg-product-specific-bioequivalence-guidance_en.pdf
38. STROMECTOL® (IVERMECTIN) TABLETS
    Oral BCS2 BCS4 1996 90s4
    https://www.accessdata.fda.gov/drugsatfda_docs/label/2008/050742s022lbl.pdf
    https://extranet.who.int/pqweb/sites/default/files/documents/21%20BE%20ivermectin_Apr2019.pdf
39. TEMODAR® (temozolomide) Capsules
    Oral BCS1 1999 90s1
    https://www.ema.europa.eu/en/documents/assessment-report/temozolomide-hospira-epar-public-assessment-report_en.pdf
40. TEMODAR® (temozolomide) for Injection

TICE® BCG BCG LIVE (FOR INTRAVESICAL USE)

41. TRUSOPT® (dorzolamide hydrochloride ophthalmic solution) Sterile Ophthalmic Solution 2%

VAQTA® [Hepatitis A Vaccine, Inactivated]

VARIVAX® [Varicella Virus Vaccine Live]

VAXELIS™ (Diphtheria and Tetanus Toxoids and Acellular Pertussis, Inactivated Poliovirus, Haemophilus b Conjugate and Hepatitis B Vaccine)

VAXNEUVANCE™ (Pneumococcal 15 valent Conjugate Vaccine) Suspension for Intramuscular Injection

42. VERQUVO™ (vericiguat) tablets, for o use
Oral BCS2 2021-2 20s2
https://www.ema.europa.eu/en/documents/assessment-report/verquvo-epar-public-assessment-report_en.pdf
43. WELIREG™ (belzutifan) tablets, for o use
Oral BCS2 2021 20s2
https://www.accessdata.fda.gov/drugsatfda_docs/nda/2021/215383Orig1s000ChemR.pdf
44. ZEPATIER® (elbasvir and grazoprevir) tablets, for o use
Oral BCS4 BCS2 2016-4 10s4
https://www.accessdata.fda.gov/drugsatfda_docs/nda/2016/208261Orig1s000ClinPharmR.pdf
45. ZERBAXA™ (ceftolozane and tazobactam) for injection, for intravenous use
46. ZINPLAVA™ (bezlotoxumab) injection, for intravenous use
47. ZOLINZA® (vorinostat capsules, for o use)
Oral BCS4 2006-4 00s4
https://www.accessdata.fda.gov/drugsatfda_docs/nda/2006/021991s000_Zolinza_ClinPharmR.pdf

Roche products:

Roche products Worldwide
https://www.roche.com/solutions/pharma/#67916b85-0b8d-44de-b762-70a48cccef3c

1. Actemra/RoActemra tocilizumab
2. Alecensa alectinib **Oral BCS2 BCS4 2015-4 10s4**
https://www.ema.europa.eu/en/documents/scientific-guideline/alectinib-hard-capsule-150-mg-product-specific-bioequivalence-guidance_en.pdf
3. Avastin bevacizumab
4. CellCept mycophenolate mofetil **Oral BCS2 BCS4 1995 90s4**
5. Copegus ribavarin **Oral BCS3 2002-3 00s3**
https://extranet.who.int/pqweb/sites/default/files/documents/BE_Ribavirin_September2021.pdf
6. Cotellic cobimetinib **Oral BCS3 2015-3 10s3**
https://www.ema.europa.eu/en/documents/assessment-report/cotellic-epar-public-assessment-report_en.pdf
7. Enspryng satralizumab
8. Erivedge vismodegib **Oral BCS2 2012-2 10s2**
https://www.ema.europa.eu/en/documents/assessment-report/erivedge-epar-public-assessment-report_en.pdf
9. Esbriet pirfenidone **Oral BCS1 2014-1 10s1**
https://www.accessdata.fda.gov/drugsatfda_docs/nda/2014/022535Orig1s000ClinPharmR.pdf
10. Evrysdi risdiplam **Oral BCS0 2020-0 20s0**
11. Fuzeon enfuvirtide
12. Gazyva/Gazyvaro obinutuzumab
13. Hemlibra emicizumab
14. Herceptin trastuzumab
15. Herceptin SC / Herceptin Hylecta trastuzumab hyaluronidase

16. Kadcyla trastuzumab emtansine
17. MabThera / Rituxan rituximab
18. MabThera SC / Rituxan Hycela rituximab hyaluronidase
19. Madopar levodopa benserazide **Oral BCS1**
    https://www.accessdata.fda.gov/drugsatfda_docs/nda/2015/203312Orig1s000ClinPharmR.pdf
20. Mircera methoxy polyethylene glycol epoetin beta
21. NeoRecormon epoetin beta
22. Ocrevus ocrelizumab
23. Pegasys peginterferon α
24. Perjeta pertuzumab
25. Phesgo pertuzumab trastuzumab hyaluronidase
26. Polivy polatuzumab vedotin
27. Pulmozyme dornase alfa
28. Roaccutane/Accutane isotretinoin **Oral BCS2 1982 80s2**
    https://www.accessdata.fda.gov/drugsatfda_docs/nda/2019/211913Orig1s000ChemR.pdf
29. Rocephin ceftriaxone
30. Ronapreve casirivimab and imdevimab
31. Rozlytrek entrectinib **Oral BCS2 2019-2 10s2**
    https://www.accessdata.fda.gov/drugsatfda_docs/nda/2019/212725Orig1s000,%20212726Orig1s000ChemR.pdf
32. Susvimo (ranibizumab injection) 100 mg/mL for intravitreal use via ocular implant
33. Tamiflu oseltamivir **Oral BCS3 1999 90s3**
    https://www.ema.europa.eu/en/documents/scientific-guideline/oseltamivir-hard-capsules-30-45-75-mg-powder-oral-suspension-6-mg/ml-12-mg/ml-product-specific-bioequivalence-guidance_en.pdf
34. Tarceva erlotinib **Oral BCS2 2004-2 00s2**
    https://www.fda.gov/files/drugs/published/N21-743S021-Erlotinib-Clinpharm-BPCA.pdf
35. Tecentriq atezolizumab
36. Vabysmo faricimab
37. Valcyte valganciclovir **Oral BCS1 2001-1 00s1**
    https://www.accessdata.fda.gov/drugsatfda_docs/label/2003/21304se1-001_valcyte_lbl.pdf
38. Xeloda capecitabine **Oral BCS0 1998 90s0**
39. Xofluza baloxavir marboxil **Oral BCS2 BCS4 2018-4 10s4**
40. Zelboraf vemurafenib **Oral BCS4 2011-4 10s4**
    https://www.accessdata.fda.gov/drugsatfda_docs/nda/2011/202429Orig1s000ClinPharmR.pdf

AstraZeneca products (https://www.astrazeneca.com/our-therapy-areas/medicines.html):

1. *Arimidex® (anastrozole) GB - oral BCS0 1995 90s0*
2. *Bambec® (bambuterol) GB oral BCS0*
3. *Bevespi® (glycopyrronium / formoterol fumarate dihydrate) GB*
4. *Bricanyl® (terbutaline sulphate) GB (also) oral BCS0 1974 70s0*

5. *Brilique® (ticagrelor)* *GB* / *NI* *oral BCS4* *2011-4* *10s4*
https://www.ema.europa.eu/en/documents/scientific-guideline/ticagrelor-film-coated-tablets-90mg-product-specific-bioequivalence-guidance_en.pdf
6. *Bydureon® (exenatide)* *GB* / *NI*
7. *Byetta® (exenatide)* *GB* / *NI*
8. *Calquence®▼ (acalabrutinib)* *GB* / *NI* *oral BCS2* *2017-2* *10s2*
https://www.accessdata.fda.gov/drugsatfda_docs/nda/2017/210259Orig1s000ChemR.pdf
9. *Casodex® (bicalutamide)* *GB* *oral BCS2* *1995* *90s2*
https://www.accessdata.fda.gov/drugsatfda_docs/label/2006/020498s018lbl.pdf
10. *Crestor® (rosuvastatin)* *GB* *oral BCS0* *2003-0* *00s0*
11. *Daxas®▼ (roflumilast)* *GB* *oral BCS2* *2011-2* *10s2*
https://www.accessdata.fda.gov/drugsatfda_docs/nda/2011/022522Orig1s000ChemR.pdf
12. *Duaklir®▼ Genuair (aclidinium/formoterol)* *GB*
13. *Eklira®▼ Genuair (aclidinium/bromide)* *GB*
14. *Fasenra®▼ (benralizumab)* *GB* / *NI*
15. *Faslodex® (fulvestrant)* *GB* / *NI*
16. *Fluenz Tetra® (live attenuated, nasal)* *GB* / *NI*
17. *Forxiga® (dapagliflozin)* *GB* / *NI* *oral BCS3* *2014-3* *10s3*
https://www.ema.europa.eu/en/documents/assessment-report/forxiga-epar-public-assessment-report_en.pdf
18. *IMFINZI®▼ (durvalumab)* *GB* / *NI*
19. *Iressa® (gefitinib)* *GB* / *NI* oral BCS2 BCS4 2003-4 00s4
https://www.ema.europa.eu/en/documents/scientific-guideline/draft-gefitinib-film-coated-tablet-250-mg-product-specific-bioequivalence-guidance_en.pdf
20. *Komboglyze® (saxagliptin/metformin hydrochloride)* *GB* / *NI* *oral BCS3 2010-3 10s3*
https://www.ema.europa.eu/en/documents/assessment-report/onglyza-epar-public-assessment-report_en.pdf
https://www.ema.europa.eu/en/documents/assessment-report/vildagliptin/metformin-hydrochloride-accord-epar-public-assessment-report_en.pdf
21. *Koselugo®▼ (selumetinib)* *GB* oral BCS4 2020-4 20s4
https://www.accessdata.fda.gov/drugsatfda_docs/nda/2020/213756Orig1s000ChemR.pdf
22. *Lokelma®▼ (sodium zirconium cyclosilicate)* *GB* / *NI* oral BCS0 2018-0 10s0
23. *Nexium® (esomeprazole)* *GB* oral BCS0 2001-0 00s0
24. *Onglyza® (saxagliptin)* *GB* / *NI* oral BCS3 2009-3 00s3
https://www.ema.europa.eu/en/documents/assessment-report/onglyza-epar-public-assessment-report_en.pdf
25. *Oxis® Turbohaler (formoterol)* *GB*
26. *Lynparza® (olaparib)* *GB* / *NI* oral BCS4 2014-4 10s4
https://www.ema.europa.eu/en/documents/variation-report/lynparza-h-c-3726-x-0016-g-epar-assessment-report-extension_en.pdf
27. *Pulmicort® (budesonide)* *GB*
28. *Qtern® (dapagliflozin/saxagliptin)* *GB* / *NI* oral BCS3 2017-3 10s3
https://www.ema.europa.eu/en/documents/assessment-report/onglyza-epar-public-assessment-report_en.pdf
https://www.ema.europa.eu/en/documents/assessment-report/forxiga-epar-public-assessment-report_en.pdf
29. *Symbicort® (budesonide/formoterol)* *GB*
30. *Synagis® (palivizumab)* *GB* / *NI*

31. *TAGRISSO®▼ (osimertinib)* GB / NI oral BCS3 2015-3 10s3
https://www.ema.europa.eu/en/documents/assessment-report/tagrisso-epar-public-assessment-report_en.pdf
32. *Trixeo® (budesonide, formoterol fumarate dihydrate, glycopyrronium)* GB
33. *Xigduo® (dapagliflozin/metformin)* GB / NI oral BCS3 2014-3 10s3
https://www.ema.europa.eu/en/documents/assessment-report/forxiga-epar-public-assessment-report_en.pdf
https://www.ema.europa.eu/en/documents/assessment-report/vildagliptin/metformin-hydrochloride-accord-epar-public-assessment-report_en.pdf
34. *Zoladex® (goserelin)* GB

Bristol Myers Squibb products:

Products on the US market.
https://www.bms.com/patient-and-caregivers/our-medicines.html

1. ABECMA® (idecabtagene vicleucel)
2. ABRAXANE® (paclitaxel protein-bound particles for injectable suspension) (albumin-bound)
3. AZACTAM® (aztreonam for injection, USP)
4. BREYANZI® (lisocabtagene maraleucel)
5. BARACLUDE® (entecavir) **oral BCS3 2005-3 00s3**
https://www.geneesmiddeleninformatiebank.nl/pars/h119701.pdf
6. CAMZYOS™ (mavacamten) **oral BCS2 2022-2 20s2**
https://www.accessdata.fda.gov/drugsatfda_docs/label/2022/214998s000lbl.pdf
7. DROXIA® (hydroxyurea) **oral BCS0 1967 60s0**
8. ELIQUIS® (apixaban) **oral BCS3 2012-3 10s3**
https://www.accessdata.fda.gov/drugsatfda_docs/nda/2012/202155Orig1s000ClinPharmR.pdf
9. EMPLICITI® (elotuzumab)
10. EVOTAZ® (atazanavir and cobicistat) **oral BCS2 2015-2 10s2**
https://www.ema.europa.eu/en/documents/assessment-report/atazanavir-mylan-epar-public-assessment-report_en.pdf
https://www.accessdata.fda.gov/drugsatfda_docs/nda/2014/203094Orig1Orig2s000ClinPharmR.pdf
11. HYDREA® (hydroxyurea) **oral BCS0 1967 60s0**
12. IDHIFA® (enasidenib) **oral BCS2 2017-2 20s2**
13. INREBIC® (fedratinib) **oral BCS0 2019-0 10s0**
14. ISTODAX® (romidepsin)
15. KENALOG®-10 (triamcinolone acetonide)
16. KENALOG® -40 (triamcinolone acetonide) and
KENALOG® -80 (triamcinolone acetonide)
17. NULOJIX® (belatacept)
18. ONUREG® (azacitidine tablets) **oral BCS3 2004-3 00s3**
https://www.accessdata.fda.gov/drugsatfda_docs/nda/2020/214120Orig1s000ChemR.pdf
19. OPDIVO® (nivolumab)
20. OPDUALAG™ (nivolumab and relatlimab-rmbw)
21. ORENCIA® (abatacept)

22. POMALYST® (pomalidomide) **oral BCS4** **2013-4** **10s4**
23. REBLOZYL® (luspatercept-aamt)
24. REVLIMID® (lenalidomide) **oral BCS3** **2005-3** **00s3**
    https://www.accessdata.fda.gov/drugsatfda_docs/nda/2005/021880s000_Revlimid_BioPharmR.pdf
25. REYATAZ® (atazanavir) **oral BCS2** **2003-2** **00s2**
    https://www.ema.europa.eu/en/documents/assessment-report/atazanavir-mylan-epar-public-assessment-report_en.pdf
26. SOTYKTU™ (deucravacitinib) **oral BCS0** **2022-0** **20s0**
27. SPRYCEL® (dasatinib) **oral BCS2** **2006-2** **00s2**
    https://www.accessdata.fda.gov/drugsatfda_docs/nda/2006/021986s000_Sprycel__ChemR.pdf
28. SUSTIVA® (efavirenz) **oral BCS2** **1998** **90s2**
    https://www.ema.europa.eu/en/documents/assessment-report/efavirenz/emtricitabine/tenofovir-disoproxil-krka-epar-public-assessment-report_en.pdf
29. THALOMID® (thalidomide) **oral BCS2 BCS4** **1998** **90s4**
    https://www.ema.europa.eu/en/documents/assessment-report/thalidomide-lipomed-epar-public-assessment-report_en.pdf
30. VIDAZA® (azacitidine)
31. YERVOY® (ipilimumab)
32. ZEPOSIA® (ozanimod) **oral BCS0** **2020-0** **20s0**
